# Cellular Uptake of Phase‐Separating Peptide Coacervates

**DOI:** 10.1002/advs.202402652

**Published:** 2024-08-30

**Authors:** Anastasia Shebanova, Quentin Moana Perrin, Kexin Zhu, Sushanth Gudlur, Zilin Chen, Yue Sun, Congxi Huang, Zhi Wei Lim, Evan Angelo Mondarte, Ruoxuan Sun, Sierin Lim, Jing Yu, Yansong Miao, Atul N. Parikh, Alexander Ludwig, Ali Miserez

**Affiliations:** ^1^ Centre for Sustainable Materials, School of Materials Science and Engineering Nanyang Technological University (NTU) 50 Nanyang Avenue Singapore 637553 Singapore; ^2^ School of Biological Sciences NTU 60 Nanyang Drive Singapore 637551 Singapore; ^3^ School of Chemistry, Chemical Engineering and Biotechnology NTU 70 Nanyang Drive Singapore 637457 Singapore; ^4^ Institute for Digital Molecular Analytics and Science (IDMxS) NTU 59 Nanyang Drive Singapore 636921 Singapore; ^5^ Departments of Biomedical Engineering and Materials Science & Engineering University of California Davis CA 95616 USA; ^6^ NTU Institute of Structural Biology NTU 59 Nanyang Drive Singapore 636921 Singapore

**Keywords:** cell uptake, drug delivery, macropinocytosis, peptide coacervates, phagocytosis

## Abstract

Peptide coacervates self‐assembling via liquid‐liquid phase separation are appealing intracellular delivery vehicles of macromolecular therapeutics (proteins, DNA, mRNA) owing to their non‐cytotoxicity, high encapsulation capacity, and efficient cellular uptake. However, the mechanisms by which these viscoelastic droplets cross the cellular membranes remain unknown. Here, using multimodal imaging, data analytics, and biochemical inhibition assays, we identify the key steps by which droplets enter the cell. We find that the uptake follows a non‐canonical pathway and instead integrates essential features of macropinocytosis and phagocytosis, namely active remodeling of the actin cytoskeleton and appearance of filopodia‐like protrusions. Experiments using giant unilamellar vesicles show that the coacervates attach to the bounding membrane in a charge‐ and cholesterol‐dependent manner but do not breach the lipid bilayer barrier. Cell uptake in the presence of small molecule inhibitors – interfering with actin and tubulin polymerization – confirm the active role of cytoskeleton remodeling, most prominently evident in electron microscopy imaging. These findings suggest a peculiar internalization mechanism for viscoelastic, glassy coacervate droplets combining features of non‐specific uptake of fluids by macropinocytosis and particulate uptake of phagocytosis. The broad implications of this study will enable to enhance the efficacy and utility of coacervate‐based strategies for intracellular delivery of macromolecular therapeutics.

## Graphical abstract

Phase‐separating peptide coacervates are promising carriers to deliver large macromolecular therapeutic inside cells, including proteins, genes, mRNA and gene editing machineries, with promising perspective for a broad range of therapeutic treatments. In this study, viscoelastic coacervate microdroplets are shown to be uptaken by cells by a non‐canonical pathway integrating features of macropinocytosis and phagocytosis.

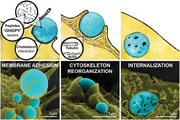

## 1 Introduction

Coacervates, or dense liquid droplets formed by liquid‐liquid phase separation (LLPS), are gaining increasing interest as novel delivery agents, including intracellular delivery.^[^ ^1^ ^]^ Both complex coacervates (when at least two oppositely charged macromolecules make the droplets) and simple coacervates (composed of single components) are being explored.^[^ ^2^, ^3^ ^]^ Promising examples recently developed by our team are the histidine (His)‐rich beak peptides –HB*pep* ^[^ ^4^, ^5^ ^]^ and HB*pep*‐SP^[^ ^6^ ^]^– derived from His‐rich proteins of the Humboldt squid (Dosidicus gigas) beak,^[^ ^7^ ^]^ which undergo LLPS to form simple coacervates at physiological conditions. The ability of HB*pep* and HP*pep*‐SP coacervate microdroplets to recruit a wide range of low molecular weight (MW) drugs and large biomacromolecules, their high transfection rates, and their low cytotoxicity at the concentration used for cell delivery represent valuable alternatives to traditional lipid‐based delivery systems.^[^ ^8^ ^]^ However, mechanistic details of the cellular uptake process are still not understood. Acquiring a comprehensive understanding of the mechanistic details involved in the cellular uptake of peptide coacervate systems is pivotal for the design and development of highly optimized peptide coacervate delivery platforms.

Cellular uptake of sub‐micron cargos is generally achieved by a variety of specialized endocytic mechanisms.^[^ ^9^, ^10^ ^]^ In contrast, larger objects (>1 µm) have limited entry routes into the cell. The two most common routes include macropinocytosis^[^ ^11^ ^]^ and phagocytosis.^[^ ^12^ ^]^ Phagocytosis mainly occurs in specialized cells (phagocytes), whereas macropinocytosis relates to the non‐specific uptake of fluid and solutes, which in general is stimulated by external stimuli, such as growth factors.^[^ ^13^ ^]^ Additionally, cell pathogens, viruses, and bacteria, whose size can vary from tens of nanometers to several micrometers, can hijack cellular uptake pathways and enter cells after engaging with the cell surface. Relevant examples include a macropinocytosis‐like mechanism, surface mimicry, and hijacking of receptor‐mediated endocytosis.^[^ ^14^, ^15^ ^]^ Such non‐canonical pathways share some features with common entry routes but are distinctly regulated.^[^ ^16^ ^]^

Because of their mesoscopic dimensions, viscoelastic deformability, and supramolecular characteristics, peptide coacervates represent a distinct class of cargo for the cell. Previous studies suggest that the cellular uptake of coacervates does not follow a singular pathway. Indeed, depending on the chemical nature, both macropinocytic route^[^ ^17^ ^]^ and direct fusion with the plasma membrane^[^ ^18^ ^]^ have been proposed. Of note, the above reports involve complex coacervates, for which charge‐charge interactions can lead to complete internalization into liposomes under certain conditions.^[^ ^19^ ^]^ Another report has demonstrated a co‐assembly of a phase‐separated peptide with a transmembrane enzyme directly on the cell membrane which led to membrane leakage.^[^ ^20^ ^]^ In contrast, the cellular uptake of single‐component peptide or protein coacervates remains largely unknown. While a recent study suggested that the uptake of coacervates made from a short arginine (Arg) ‐containing peptide is cholesterol‐dependent and might involve lipid rafts due to sensitivity to methyl‐β‐cyclodextrin (MβCD), the exact mechanism has not been studied in detail.^[^ ^21^ ^]^ HB*pep* and HB*pep*‐SP peptides are simple coacervates that are enriched not only in His but also in tyrosine (Tyr) residues (**Table** **1**), in addition to bearing one tryptophan (Trp) at the C‐terminus.^[^ ^5^ ^]^ The difference between HB*pep* and HB*pep*‐SP is that the latter has one additional lysine (Lys) residue conjugated with a side chain containing a disulfide bond and a self‐immolative moiety. The side chain plays a central role in the therapeutic release as upon interaction with endogenous glutathione, it is cleaved from the backbone resulting in the disassembly of the coacervates and, thus, payload release.^[^ ^6^ ^]^ Owing to its terminal benzoate group at the end of the pendant side chain, HB*pep*‐SP is more hydrophobic than HB*pep*. In addition to electrostatic interactions, other types of interactions may be involved to bind to the cell membrane. In particular, aromatic residues often enriched in simple coacervates tend to be present in the interfacial region of lipid membranes^[^ ^22^ ^]^ and are usually involved in protein/lipid bilayer interactions^[^ ^23^ ^]^ including the interaction with cholesterol^[^ ^24^ ^]^ and peptide/protein membrane anchoring.^[^ ^25^ ^]^ Accordingly, tuning the properties of aromatic rings has increased the cellular uptake of peptides.^[^ ^26^ ^]^ A recent study of HB*pep* interacting with lipid bilayer at physiological pH indicates that the peptide tends to associate with the membrane surface without disturbing the bilayer, and that the aromatic amino acids such Tyr and Trp, as well as His, can interact with β‐hydroxy group of cholesterol.^[^ ^27^ ^]^ This suggests that cholesterol‐peptide interactions might play a key role in coacervate membrane association, and thus cell uptake, although further investigations are needed to confirm this.

**Table 1:** Amino acid sequences of HBpep and HBpep‐SP peptides.

I.D	Sequence	Number of Residues	Modification
HBpep	GHGVY GHGVY GHGPY GHGPY GHGLYW	26	none
HB*pep*‐SP	GHGVY GHGVY GHGPY Kx GHGPY GHGLYW	27	Lysine ε‐amine (residue # 16)

In the work reported here, we show that HB*pep* and HB*pep*‐SP coacervates are taken up by a mechanism combining defining features of both macropinocytosis and phagocytosis. Our studies with simple model membrane systems such as Giant Unilamellar Vesicles (GUVs) revealed that coacervates attach to the membrane surface but do not enter the lumen. Live cell time‐lapse imaging experiments showed dynamic progressive membrane engulfment during the uptake process. Further experiments exploiting direct visualization of cellular uptake of coacervates by transmission and scanning electron microscopy (TEM and SEM) allowed us to directly visualize the membrane engulfment and endocytosis of coacervates in two different cell lines, HeLa and HepG2. We captured different stages of HB*pep* and HB*pep*‐SP coacervates internalization, from attachment to membrane wrapping to complete membrane engulfment. At the attachment stage, the involvement of the cell's filopodia in capturing the coacervates was observed, which is likely a critical step toward their subsequent internalization. Quantitative Fluorescence Activated Cell Sorting (FACS) analysis in the presence of chemical inhibitors targeting cell cytoskeleton revealed the critical role of formin‐mediated actin polymerization during cell uptake. In addition, modulating the adhesion of coacervates to the cell membrane by recruiting cholesterol‐binding peptides within the coacervates enhanced cell uptake. This study provides a new understanding of the interaction of His‐ and aromatic‐rich phase‐separating peptide coacervates with model membranes and their subsequent cell uptake via actin and cholesterol‐dependent, non‐classical endocytosis.

## 2 Results and Discussion

### 2.1 Interaction Between HB*pep* and HB*pep*‐SP Coacervates and GUVs: Roles of Membrane Charge and Fluidity

**Figure 1:**
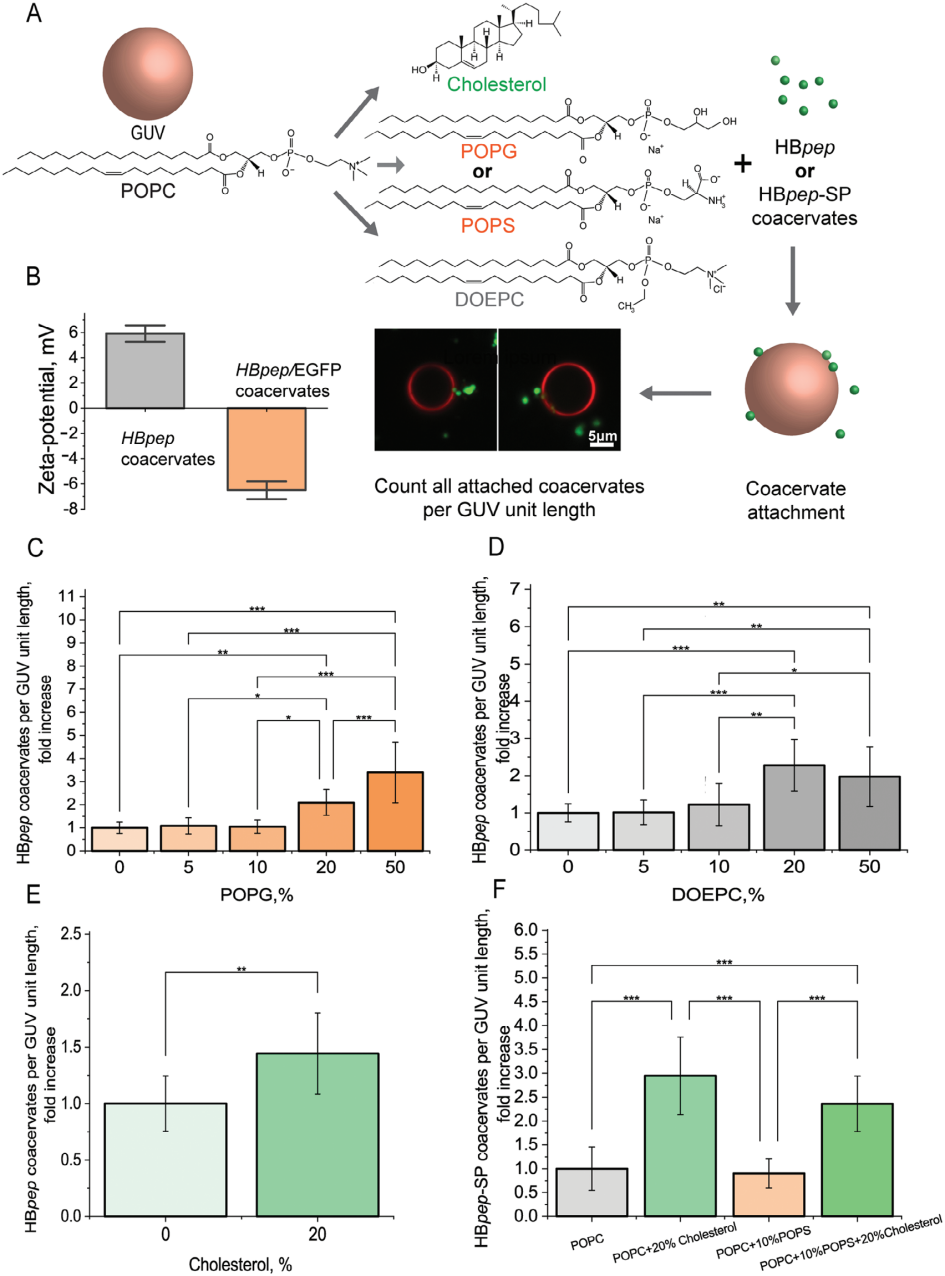
Interaction between HB*pep* and HB*pep*‐SP coacervates and GUVs. A) Schematics of the experimental setup for studying the attachment of coacervates to GUVs with varying lipid composition. The attachment was monitored using fluorescent imaging. B) Zeta‐potential of HB*pep* and HB*pep* coacervates loaded with EGFP. C, D) Plots of HB*pep* coacervate attachment to POPC GUVs with increasing amounts of negatively charged POPG (C) and positively charged DOEPC (D) showing that coacervate attachment increases when the content of charged lipids increases in GUVs. E) Plot of attachment of HB*pep* coacervates to POPC GUVs with and without 20% cholesterol indicates that coacervate attachment is higher for POPC GUVs with 20% cholesterol. Data are presented as the mean ± SD, *N* = 10, (*N*: number of GUVs). F) Plot of attachment of HB*pep*‐SP coacervates to POPC GUVs with and without 10% POPS and 20% cholesterol indicating higher attachment with 20% cholesterol. Data were normalized against baseline attachment to POPC GUVs and are presented as fold increase, mean ± SD, *N* = 8 (*N*: number of fields of view). One‐way ANOVA was used to compare the groups, * = *P* < 0.05, ** = *P* < 0.01, *** = *P* < 0.001.

Cellular uptake of most nano‐ or microparticles begins with their adhesion to the outer leaflet of the plasma membrane and is often dictated by electrostatic interactions. To determine whether membrane lipid charge influences the initial step of HB*pep* coacervates adhesion, HB*pep* coacervates entrapping Enhanced Green Fluorescence Protein (EGFP) were mixed with GUVs prepared from the zwitterionic phospholipid 1‐palmitoyl‐2‐oleoyl‐glycero‐3‐phosphocholine (POPC), as well as with an increasing amount of positively or negatively charged phospholipids (**Figure** **1**A, chemical structure). GUVs are simplified model membrane systems with tunable physio‐chemical properties, making them a convenient tool for studying membrane‐coacervate interactions.^[^ ^28^ ^]^ HB*pep* is a zwitterionic peptide with charge contributions from five His residues in its sequence and two oppositely charged termini (Table 1), resulting in a theoretical pI of 7.97. Thus, the coacervates should be partially positively charged at pH 7.5. The measured zeta‐potential was slightly positive, 5.9 ± 0.6 mV, but turned slightly negative, −7.1 ± 0.2 mV, after loading the coacervates with EGFP (Figure 1B). Mixing HB*pep* coacervates with POPC GUVs containing increasing amounts of either positively or negatively charged phospholipids showed that HB*pep* coacervates bound more to GUVs with higher content of charged lipids, but never crossed the GUV lumen (Figure 1C, D; Figures S1 and S2, Supporting Information). In general, we observed that coacervate adhesion to membranes increased with the charged lipids regardless of the type of charge. Coacervate attachment increased with an increasing amount of negatively charged 1‐Palmitoyl‐2‐oleoyl‐sn‐glycero‐3‐phospho‐rac‐(1‐glycerol) (POPG), attaining a maximum of 3.4 (±1.3) fold increase in attachment when the POPG concentration was 50 mol% (Figure 1C). Notably, coacervate attachment also increased with an increasing amount of 1,2‐dioleoyl‐sn‐glycero‐3‐ethylphosphocholine (DOEPC), with a 2.28 (± 0.69) fold increase at the DOEPC concentration of 20 mol% and 1.97 (± 0.8) fold increase in attachment at the DOEPC concentration of 50 mol.% (Figure 1D). Despite the coacervates loaded with EGFP having a negative zeta potential (due to the net charge of EGFP), they could bind to both positively and negatively charged GUVs. While electrostatic interactions are obviously important, aromatic‐rich peptides add complexity to membrane/peptide coacervate interactions. Andreev et al.^[^ ^29^ ^]^ showed that hydrophobic interactions might govern the adhesion of aromatic‐rich peptoids to charged lipid bilayers, with high aromatic content or membrane charge leading to a stronger interaction with the bilayer and even its disruption. Therefore, in the case of HB*pep* coacervates, their preference for charged membranes might also be related to their high aromatic content (23 mol.%). Note that the outer layer of the mammalian cell plasma membrane mainly consists of zwitterionic lipids,^[^ ^30^ ^]^ which may not result in interactions as strong as that with the highly charged GUVs.

To evaluate the role of membrane cholesterol level on coacervate attachment, POPC GUVs containing 10 to 50 mol.% cholesterol were incubated with HB*pep* coacervates (Figure 1A). Cholesterol is an integral component of eukaryotic cell membranes and is a key molecule controlling membrane fluidity. Cholesterol concentration within the cell membranes can range anywhere from <5 mol% in mitochondrial membranes to >25 mol% in plasma membranes.^[^ ^30^ ^]^ Interestingly, HB*pep* coacervates exhibited an appreciable increase in attachment only for POPC GUVs containing 20 mol% cholesterol (1.44 ± 0.36  fold increase in attachment, Figure 1E; Figures S3 and S4, Supporting Information), whereas the attachment to POPC GUVs containing 10, 30, 40 and 50 mol% cholesterol decreased (Figure S4, Supporting Information). At higher cholesterol levels (above 30%), coacervate attachment decreased to below 0.7‐fold, suggesting that excessive membrane rigidity was unfavorable for coacervate attachment. In a study on large unilamellar POPC vesicles, no significant changes in bending rigidity were observed up to 20 mol% cholesterol, whereas higher cholesterol levels resulted in a meaningful stiffness increase.^[^ ^31^ ^]^ Thus, at higher cholesterol levels, the energy cost of membrane bending might be too high and not counterbalanced by the increase in adhesion energy of coacervates. Additional experiments with lipid rafts spontaneously induced on GUVs using a 40% POPC/40% sphingomyelin (SM)/20% cholesterol lipid composition did not show a preferred attachment of coacervates to a more ordered phase. Indeed, they were mostly attached to the less‐ordered parts of GUVs (Figure S5, Supporting Information). It is also possible that a specific range of fluidity modulated by cholesterol is favorable to anchor the aromatic amino acids to the cell membrane, thus facilitating coacervate attachment, or that they even have some affinity to cholesterol. We also assessed the interaction of HB*pep*‐SP coacervates on GUVs and confirmed enhanced attachment (2.95 ± 0.81 fold increase) with 20% cholesterol content in GUVs (Figure 1F; Figure S6, Supporting Information). The addition of negatively charged 1‐palmitoyl‐2‐oleoyl‐sn‐glycero‐3‐phospho‐L‐serine (POPS) – a component presented in native cell membranes^[^ ^30^ ^]^ and used here at a physiologically relevant concentration – to POPC GUVs did not significantly alter the coacervate attachment, showing only 0.9 ± 0.3‐fold change. In contrast, incorporating 20% cholesterol to POPC/POPS containing GUVs markedly enhanced the attachment, with an increase of 2.4 ± 0.6‐fold (Figure 1F). While the above results indicate that a certain cholesterol content is preferred for coacervate attachment, the latter did not cross the membrane into the GUV lumen independent of the cholesterol concentration.

### 2.2 Cellular Uptake of HB*pep* and HB*pep*‐SP Coacervates Visualized by Live Cell Imaging

**Figure 2:**
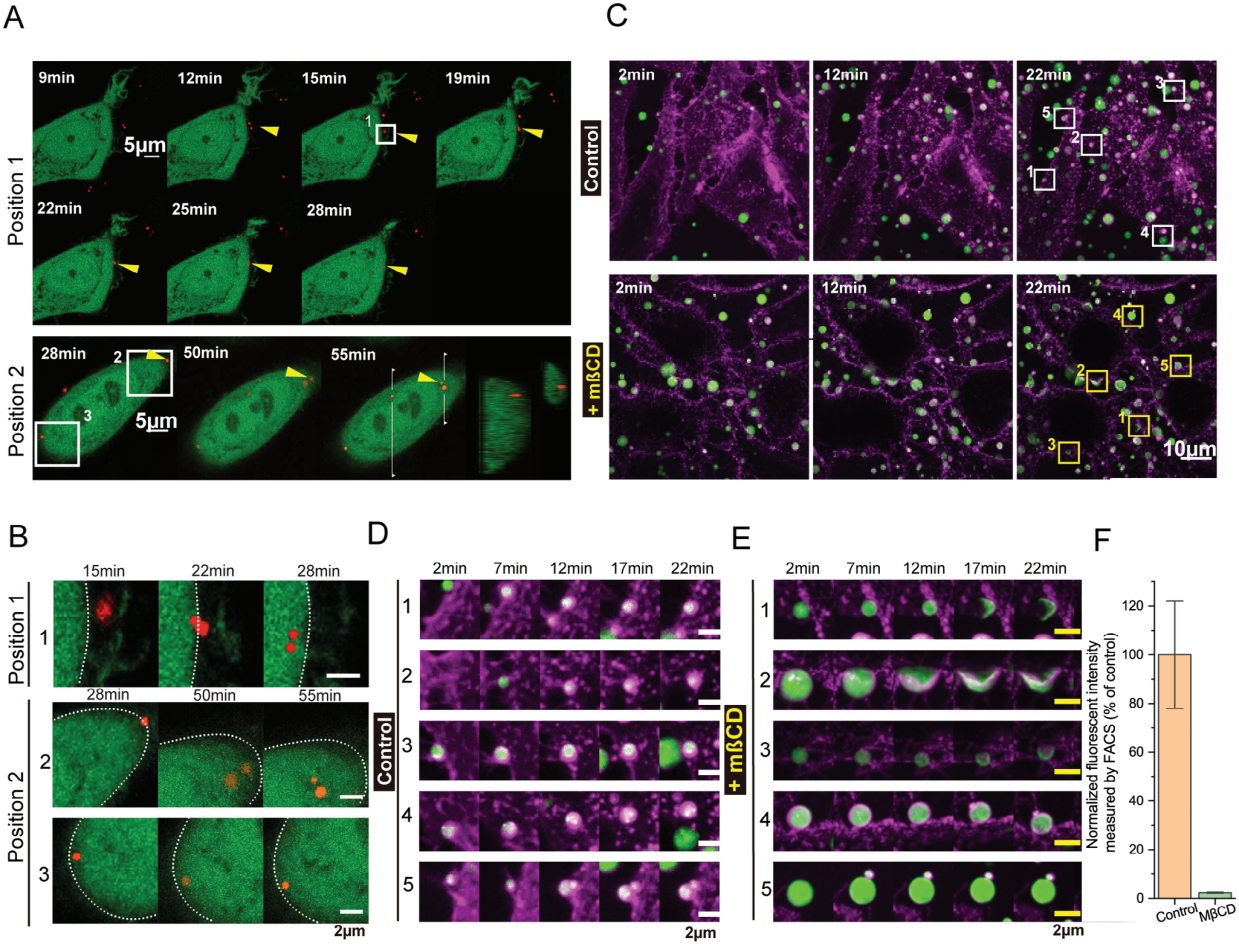
Live cell imaging of HB*pep* and HB*pep*‐SP coacervate uptake. A) Interaction of GFP‐expressing HeLa cells with mCherry‐loaded HB*pep* coacervates demonstrates the attachment of coacervates (yellow arrowheads) to the cell, their colocalization with cell protrusions during uptake, and inward movement after the internalization. The cross‐section of the reconstructed stack in the yz plane (panel A, position 2) confirms the internalization. B) Enlarged vie ws of areas selected on images from panel A. C) Time‐lapse confocal images of cellular uptake of HB*pep*‐SP coacervates incubated with HeLa cells stained with cell mask membrane dye without (top) and with (bottom) MβCD treatment, demonstrating the progressive engulfment only without MβCD. D, E) Closeup views of coacervates selected on the images from panel (C) for the control (D) and in the presence of 5 mM MβCD (E). Magenta – Cell Mask Deep Red dye, green – EGFP. F) FACS analysis of HB*pep*‐SP coacervate uptake in the presence or absence of 5 mM MβCD (for cells in the presence of MβCD, 50 min of MβCD pre‐treatment was applied).

To directly visualize the initiation of internalization of HB*pep* and HB*pep*‐SP coacervates at the plasma membrane, we performed live cell imaging experiments using a high‐speed spinning disk confocal microscope. First, we investigated the uptake of mCherry‐loaded HB*pep* coacervates in GFP‐expressing cells to visualize the internalization. In the time‐lapse image series, we observed that upon addition to cells, the coacervates were floating around and randomly settled on the cell surface (**Figure** **2**A, arrowheads). In some cases, cell protrusions that extended from the cell surface were observed (Figure 2A, position 1) and seemed to be involved in the capture of coacervates (Figure 2B, position 1, Figure S7, Supporting Information). Upon settling on the cell surface, many coacervates were internalized by the cells (Figure 2A, position 2), moved toward the intracellular space (Figure 2B, position 2, panel 2), and co‐existed with coacervates attached to the plasma membrane but that did not move inwards even 50 min after adding the coacervates (Figure 2B, position 2, panel 3). To visualize the coacervate‐plasma membrane interactions in more detail, we observed the cellular uptake of EGFP‐loaded coacervates in HeLa cells stained with the Cell Mask Deep Red membrane dye (Figure 2C–E). The uptake process began with the attachment of the coacervate to the cell surface, followed by progressive engulfment, and culminated with complete cell uptake, as shown in our acquired images taken every 5 mins (Figure 2C, upper panel, and 2D, coacervates 1–4). Many coacervates were fully engulfed and internalized during the observed period, although some showed signs of partial engulfment (Figure 2D, coacervate 5).

Next, we monitored the association and internalization of coacervates in cholesterol‐depleted cells, which was achieved by treating the cells with the cholesterol‐depleting molecule MβCD (5 mM for 50 min prior to the addition of coacervates). Strikingly, this treatment completely abolished uptake, with the membrane unable to fully engulf the coacervates (Figure 2C,E). Some coacervates (≈1 in 10) changed their shape and partially wetted the cell surface (Figure 2E, coacervates 1,2). It should be mentioned that the cell mask dye was partially adsorbed by the coacervates, resulting in irregular fluorescent signal pattern at the plasma membrane/coacervates interface. Nevertheless, the inability of coacervates to cross the cell membrane remained clearly visible. To quantify and compare the efficiency of uptake of coacervates with and without MβCD, we conducted FACS experiments after washing away non‐internalized coacervates. The uptake rate in the presence of MβCD was only 2.3 ± 0.2% compared to the uptake without MβCD, showing that the latter essentially abrogates coacervate uptake (Figure 2F).

### 2.3 TEM Studies of Cellular Uptake of HB*pep* and HB*pep*‐SP Coacervates in HeLa and HepG2 Cells

**Figure 3:**
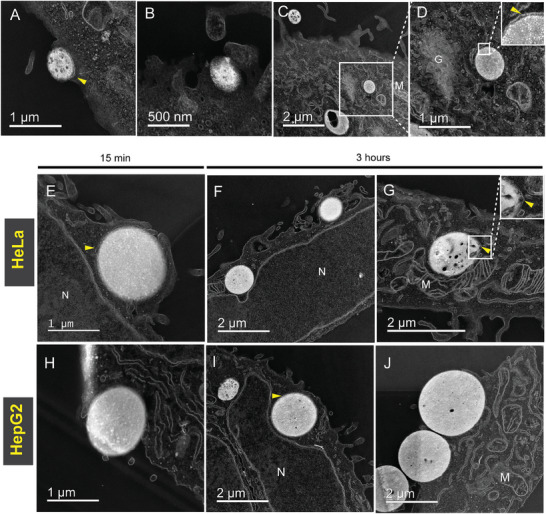
The uptake of HB*pep* and HB*pep*‐SP coacervates in HeLa and HepG2cells visualized by TEM. A–D) Ultrathin sections of fixed and resin embedded HeLa cells showing HB*pep* coacervates during different stages of cell uptake. (A,B) Different stages of membrane engulfment around the droplet. (C,D). Fully internalized coacervate in the cell cytoplasm. E–G) Ultrathin sections of fixed and resin embedded HeLa cells showing HB*pep*‐SP coacervates during the various stages of cell uptake after 15 min (E) and 3 h (F,G) of incubation. H–J) Ultrathin sections of fixed and resin embedded HepG2 cells showing HB*pep*‐SP coacervates during the various stages of cell uptake after 15 min (H) and 3 h (I,J) of incubation. N – nucleus, M – mitochondrion, G – Golgi complex. The observed membrane around the coacervates is indicated with arrows. Additional images are provided in Figures S9 and S10 (Supporting Information).

To visualize the coacervate/membrane interaction and internalization process at higher resolution, ultrathin (70–100 nm) sections of fixed and resin‐embedded HeLa cells treated with HB*pep* coacervates for 3 h were imaged using the High‐Angle Annular Dark‐Field Scanning TEM (HAADF‐STEM) technique. **Figure** **3**A–D is a panel of HAADF‐STEM images that show micron‐sized spherical droplets, representing HB*pep* coacervates, at various stages of the uptake process: partial membrane engulfment (Figure 3A), at an advanced stage of cup‐shaped extensions whose leading rims appear to close over the coacervates (Figure 3B), and internalized coacervates in the cell cytoplasm (Figure 3C,D). Since HAADF‐STEM is a dark‐field technique where the objects containing electron‐dense elements appear brighter on the image, cell membranes stained with osmium tetroxide (OsO_4_) appear bright on a darker background. The high electron density observed for the coacervates is likely due to the high peptide concentration within the coacervates and heavy metal enrichment during staining. For this experiment, we also loaded the coacervate with ferritin^[^ ^32^ ^]^ in addition to EGFP. Ferritin iron cores appeared as bright dots within the coacervates (**Figure** **3**A–D) and helped us to distinguish them from other electron‐dense structures like lipid droplets. Notably, coacervates exhibited an inhomogeneous and porous internal structure which might be explained by the presence of gel‐like domains within the coacervates. Liquid‐solid coexistence has been shown for other types of protein coacervates and is attributed to aging,^[^ ^33^, ^34^ ^]^ which may also occur in HB*pep* and HB*pep*‐SP coacervates. The gel‐like nature of coacervates was further confirmed by Fluorescence Recovery After Photobleaching (FRAP) experiments in which HB*pep* coacervates demonstrated only 20% recovery 10 min after addition of coacervates to the Optimem media, and no recovery after 1 h (Figure S8A–C, Supporting Information). Besides being electron‐dense, some coacervates were slightly elongated (Figure 3C), which is expected given their deformability. Additionally, fully membrane‐enclosed coacervates were identified within the cytoplasm (Figure 3A,C,D, yellow arrowhead). The cup‐shaped plasma membrane structures identified in Figure 3A,B are a characteristic feature of macropinocytosis and phagocytosis^[^ ^13^ ^]^ that involves actin polymerization and reorganization. In general, the process of macropinocytosis is morphologically very similar to that of phagocytosis. However, there are notable differences. The engulfment process during phagocytosis is driven by the protrusive force generated by actin polymerization. It is initiated by the adhesion of cargo particles to the membrane through specific receptor interactions and works against the surface tension related to membrane and cortical tension.^[^ ^35^, ^36^ ^]^ Macropinocytosis, on the other hand, relies on flat membrane protrusions that occasionally fold back to form circular ruffles, even in the absence of any particular cargo. The resulting macropinosomes usually contain some liquid surrounding internalized particles. By contrast, a tight sleeve is formed around an internalized object during phagocytosis. In the case of HB*pep* coacervates, we observed a connection between the membrane and the surface of internalized coacervates, which is more characteristic of phagocytosis (Figure 3D). Specifically, the tighter adhesion of coacervates to the lipid bilayer may be because the microdroplets are enriched in aromatic amino acids and their interaction with lipids.

We further extended our studies to HB*pep*‐SP peptide coacervates to HeLa and HepG2 cells (Figure 3E–J). Unlike HB*pep* coacervates, the HB*pep*‐SP coacervates undergo disassembly once inside the cells, triggered by the reduction of the disulfide bond located in the moiety attached to the Lys residue, with concomitant release of the payload.^[^ ^6^ ^]^ Similar to HB*pep* coacervates, multiple electron‐dense coacervates were observed, demonstrating membrane engulfment and endocytosis in both cell lines (Figure 3E–J). Micrometer‐sized droplets attached to the cell membrane, as well as fully internalized droplets within the cells, could be seen after 15 min (Figure 3E,H; Figure S9, Supporting Information) and 3 h (Figure 3F,G,I,J) of incubation. Since HB*pep*‐SP coacervates disassemble once inside the cell, we observed some signs of this disassembly process (Figure 3G; Figure S10, Supporting Information) in the form of incipient pores in the internalized coacervates. Additionally, some droplets appeared to be “sinking” into the cell, thereby bending the cell membrane (Figure 3J; Figure S9, Supporting Information). The inward movement of an engulfed object has been observed for phagocytosis, and its nature is still debated. It has been suggested to result from a balance between tension in the membrane (leading to cell rounding) and the resistance of cytoplasmic viscosity to the cell shape change.^[^ ^37^ ^]^ On the other hand, the phagocytic cup formation and sinking behavior might also be related to distinct regulation mechanisms. Walbaum et al. showed that deletion of the tyrosine protein kinase SYK in macrophages did not impair the sinking complement‐mediated phagocytosis, but abolished the Fcγ receptor‐mediated uptake through phagocytic cup formation.^[^ ^38^ ^]^ Interestingly, a recent study demonstrated a sunken morphology for the phosphatidylserine (PS)‐mediated phagocytosis of apoptotic cells.^[^ ^39^ ^]^ In the case of coacervates, we observed the combination of sinking and protrusive behavior in both HeLa and HepG2 cells.

Some thin filaments around the coacervates could be observed (Figure 3E,H; Figure S9A–F, Supporting Information), which probably represent filopodia. We could not visualize filopodia for HB*pep* and HeLa cells (Figure 3A–D) because the sections were prepared from a cell monolayer sectioned parallel to the substrate surface. In contrast, HB*pep*‐SP cell samples were prepared as a pellet, resulting in multiple cell orientations from the section that could be imaged. In comparison to HB*pep* coacervates, HB*pep*‐SP looked less deformed by the cytoskeleton. As mentioned above, HB*pep*‐SP is more hydrophobic due to the conjugated sidechain, and more hydrophobic peptides have been shown to exhibit a higher storage modulus *G*′, making them less deformable.^[^ ^40^ ^]^ Additionally, HB*pep*‐SP coacervates demonstrated no recovery in FRAP experiments performed in Optimem media (Figure S8D, Supporting Information) suggesting their fast gelation. We attempted to perform nanoindentation by liquid cell Atomic Force Microscopy (AFM) to assess the viscoelastic properties of HB*pep* and HB*pep*‐SP coacervates. For HB*pep* coacervates, moduli measurements could not be performed since the coacervates fully spread onto the mica surface owing to their high wettability (Figure S11A, Supporting Information). In the case of HB*pep*‐SP coacervates, although the coacervates were slightly flattened on the mica surface (Figure S11B, Supporting Information), their shape was partially preserved, enabling to obtain force‐displacement curves (Figure S11C, Supporting Information) that were subsequently fit with the Hertzian model. Interestingly, the average Young modulus of 10.31 ± 6.78 kPa (Figure S11D, Supporting Information) is well within the proposed mechano‐sensitivity range of living cells,^[^ ^37^ ^]^ suggesting that mechano‐sensing could also influence coacervate uptake.

### 2.4 Characterizing the Spatial Relationship between HB*pep* and HB*pep*‐SP Coacervates and Plasma Membrane with SEM

**Figure 4:**
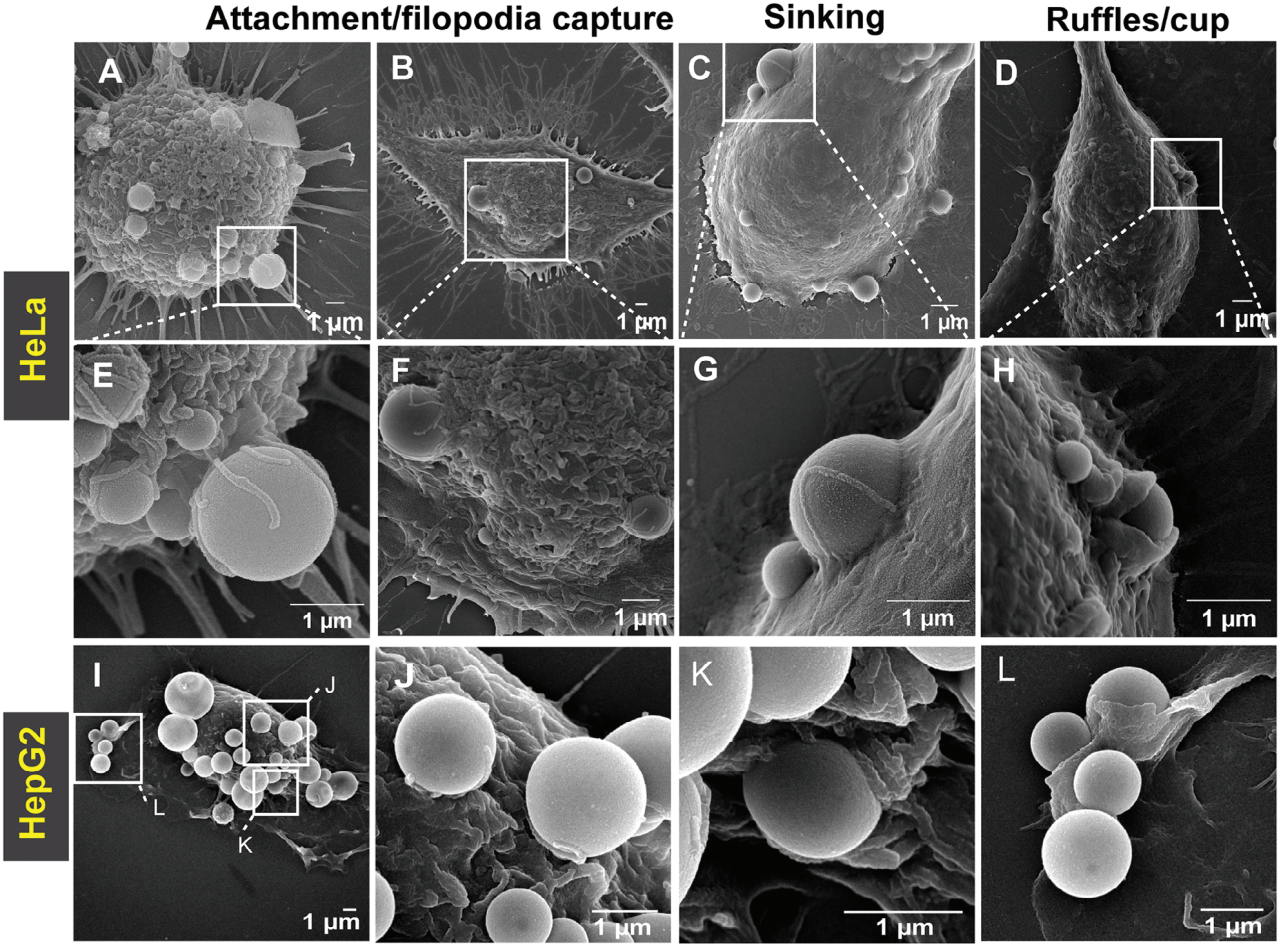
SEM images of HB*pep* and HB*pep*‐SP coacervates interacting with HeLa and HepG2 cell surface showing various stages of uptake. A–D) Representative SEM micrographs of HeLa cells with multiple HB*pep* coacervates after 15 min of incubation. E‐H. Close‐up views from A–D illustrate the three main types of coacervates/cell interaction topologies. Adhesion and filopodia capture E, F), sinking G), and ruffle/cup H). I) Representative SEM micrograph of HepG2 cell with multiple HB*pep*‐SP coacervates after 15 min of incubation. J–L) Close‐up views illustrating the three main coacervates/cell interaction topologies of coacervates from (I). Additional images are provided in Figures S12–S14 (Supporting Information).

Since TEM is not suitable for providing high‐resolution topological information of the cell surface, we opted for SEM to gain further insights into the attachment and internalization process. **Figure** **4** is a panel of representative SEM images that show HB*pep* coacervates attached to the surface of HeLa cells (Figure 4A–H) and HB*pep*‐SP coacervates on the surface of HepG2 cells after 15 min of incubation (Figure 4I,L). Multiple coacervates were observed interacting with the cell surface (Figure 4A–D,I), and some were attached to the cell and surrounded by filopodia (Figure 4A,B,E,F,I,J; Figure S12, Supporting Information). We also observed flat membrane extensions wrapping coacervates (Figure 4L) as well as cup‐shaped structures surrounding the coacervates (Figure 4H). Closer inspection of SEM images for both HB*pep* and HB*pep*‐SP in HeLa and HepG2 cell lines revealed different stages of membrane engulfment (Figure 4; Figure S13A–L, Supporting Information), in agreement with our TEM data. We classified the observed coacervate/cell interaction topologies as “adhesion”, “sinking” (Figure 4C,G,K; Figure S14, Supporting Information) and “ruffle/cup”. The wrapping of coacervates by filopodia has an intriguing parallel with filopodia capture of some pathogens during the initial stages of attachment to the cell.^[^ ^16^ ^]^ SEM did not allow us to visualize fully internalized coacervates, although we could see some bumps on the cell surface (Figure S15, Supporting Information) that may represent fully engulfed coacervates that are in the process of being internalized. In addition, since filopodia are mechanosensitive structures,^[^ ^41^, ^42^ ^]^ these observations suggest that the uptake process is mechanosensitive.

### 2.5 Actin Cytoskeleton‐Coupled Morphogenesis Mediates Coacervates Uptake

Having confirmed that membrane engulfment and filopodia play a role in coacervate capture (Figure 2A,B; Figure 4; Figures S9, S10 and S12–S14, Supporting Information), we hypothesized that the uptake process is cytoskeleton‐dependent. In our previous experiments, disrupting actin filaments by the actin barbed‐end inhibitor cytochalasin B did not appear to affect uptake.^[^ ^5^ ^]^Similarly, theoretical predictions supported by experiments with cytochalasin D‐treated cells indicate that membrane engulfment during phagocytosis is possible, although not entirely efficient, in the absence of a fully functional actin cytoskeleton, and can result in the formation of half‐engulfed cups.^[^ ^43^ ^]^ Moreover, cytochalasin D also showed subsequent effects in altering cell membrane cortical tension^[^ ^44^ ^]^ and can facilitate the diffusion of receptors.^[^ ^45^ ^]^ To avoid the potential underestimation of F‐actin in coacervates uptake when using specific barbed‐end inhibitors, we used well‐known small molecule inhibitors targeting various steps of actin cytoskeleton assembly. We focused on HB*pep*‐SP peptide for these studies owing to its potential application as a drug delivery agent and quantified coacervates uptake using FACS.

**Figure 5:**
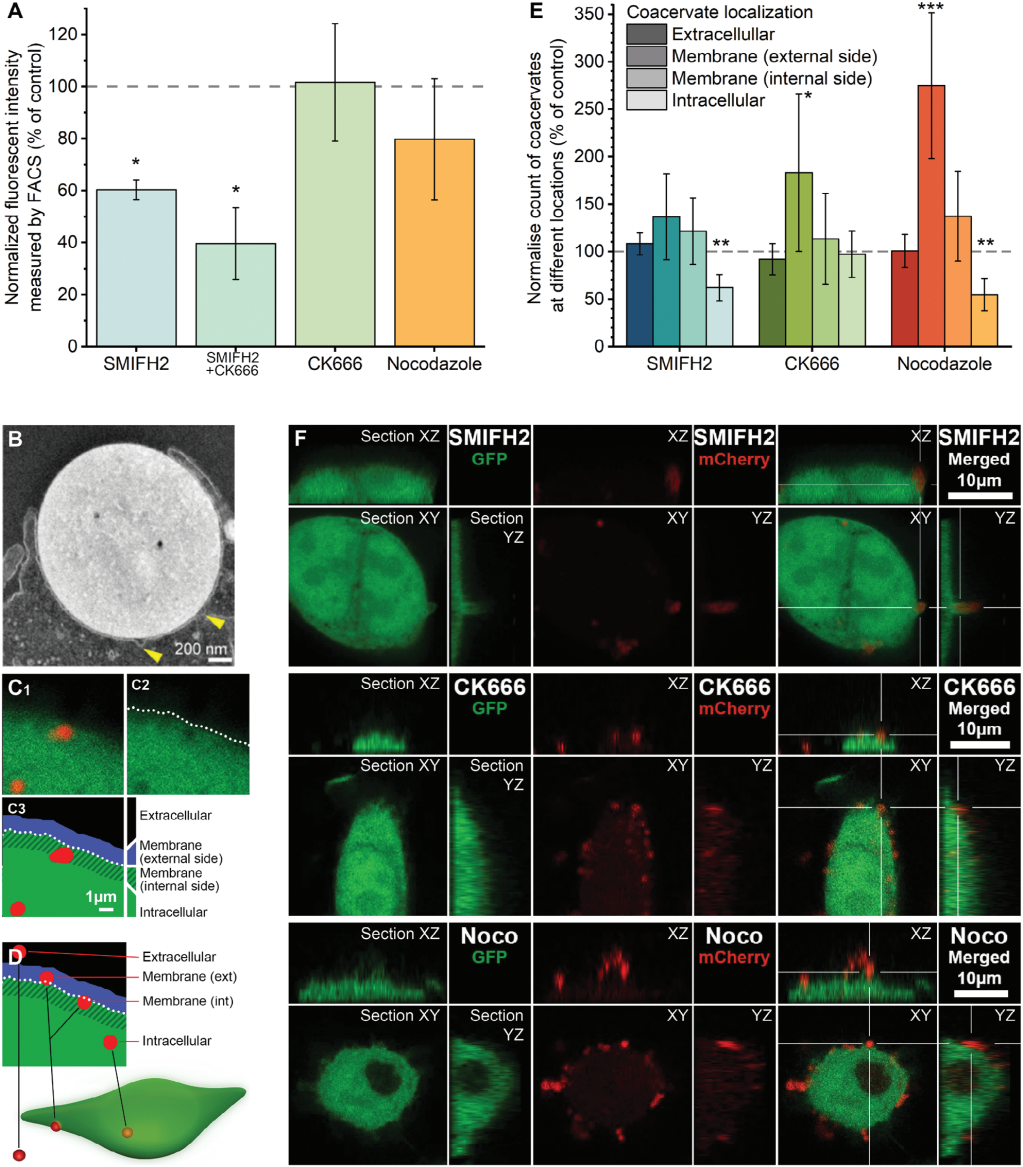
Cell cytoskeleton during coacervate uptake. A) FACS analysis of mCherry‐loaded HB*pep*‐SP coacervate uptake by GFP‐expressing HeLa cells in the presence of SMIFH2 (20 µM), CK666 (100 µM), and Nocodazole (10 µg ml^−1^). The data were normalized to control (no inhibitor added) and are shown as a mean ± SD, *N* = 3. B) TEM image showing vesicle fusion at the coacervate attachment site suggests the delivery of endomembranes (yellow arrows). C, D) 3D confocal imaging processed with CellProfiler to segment cell volume (using GFP channel) and coacervates (red mCherry channel). All coacervates were counted in different locations: extracellular, at the membrane (outside or inner side) and intracellular. C. Zoom‐in of a confocal slice (C_1_), threshold‐based membrane detection (C_2_), and 1 µm wide external and internal side of the membrane (C_3_). D) Schematic of coacervates at different locations in the cell. E) Number of coacervates at different locations normalized to the controls and shown as the mean ± SD, *N* = 8. F. Representative sections of HeLa‐GFP cells incubated with mCherry‐loaded coacervates in the presence of SMIFH2, CK666 and Nocodazole inhibitors. XY, XZ and YZ sections are shown for each inhibitor. Two sample *t*‐test was used to compare the groups with the controls. * = *P* < 0.05, ** = *P* < 0.01, *** = *P* < 0.001.

In previous studies, the pinocytosis inhibitor amiloride did not significantly affect the cellular uptake of coacervates.^[^ ^4^, ^5^ ^]^ Here, we used its more potent derivative EIPA^[^ ^46^ ^]^ (100 µM), which inhibits macropinocytosis, and observed a decrease in cellular uptake of coacervates loaded with the red fluorescence protein mCherry (Figure S16, Supporting Information), chosen in order to differentiate coacervates from EGFP‐expressing cell in associated imaging experiments. Of note, the filopodia capture phenomenon was also observed for the chosen conditions (Figure S17, Supporting Information). We next thought to delineate which effectors of actin polymerization play a role in coacervate uptake. Applying 20 µM of SMIFH2^[^ ^47^ ^]^ significantly reduced the uptake of coacervates in both EGFP‐expressing HeLa (**Figure** **5**A) and HeLa cells (Figure S16, Supporting Information). SMIFH2 inhibits formin‐mediated actin polymerization^[^ ^47^ ^]^ as well as dimer‐dimer interactions of formins,^[^ ^48^ ^]^ which generate unbranched (linear) actin filaments in filopodia‐like protrusions.^[^ ^49^ ^]^ This mechanism thus appears to be consistent with the involvement of protruding structures surrounding coacervates during initial invagination observed by both fluorescent imaging and SEM (Figure 4). Compared to the formin inhibitor, treating HeLa‐GFP cells with 100 µM CK666,^[^ ^50^ ^]^ which blocks the nucleation of branched actin networks by inhibiting the Arp2/3 complex, did not seem to affect coacervate uptake (Figure 5A; Figure S16, Supporting Information). However, a combination of SMIFH2 and CK666 reduced even further the uptake compared to SMIFH2 alone, indicating that branched actin may amplify the dominant role of linear actin polymerization during capture and internalization of coacervates (Figure 5A), Formins and the Arp2/3 complex have been shown to play distinct roles in actin polymerization during phagocytosis in macrophages.^[^ ^51^ ^]^ Inhibition of the Arp2/3 complex with CK666 decreases surface ruffling, resulting in particle uptake reminiscent of the sinking behavior, whereas blocking formins with SMIFH2 has been linked to excessive ruffling.^[^ ^51^ ^]^ Possibly, both the Arp2/3 complex and formins are involved in specific aspects of actin polymerization during cellular uptake of coacervates in non‐phagocytic cells. Treating the cells with nocodazole (10 µg ml^−1^), a drug that inhibits microtubule polymerization,^[^ ^52^ ^]^ slightly affected coacervate uptake in EGFP‐HeLa cells according to FACS data (Figure 5A), an effect that was more pronounced and significant in HeLa cells (Figure S16, Supporting Information). The influence of nocodazole might be related to the function of microtubules as delivery tracks for essential components (*e.g*., endomembranes) to the uptake site, which is consistent with TEM images where membrane vesicles were seen to fuse at the uptake site (Figure 5B; Figure S18, Supporting Information).

The difference in cell uptake between HeLa and GFP‐HeLa cells may be explained by the inability of FACS to unambiguously distinguish between attached, partially engulfed and fully internalized coacervates. Therefore, a complementary method was developed to discriminate whether the coacervates were localized outside the cell, bound to the membrane (both the outside and the inner cell membrane), or fully internalized (Figure 5C–E; Figure S19, Supporting Information). The method consisted of incubating EGFP‐HeLa cells with mCherry‐loaded HB*pep*‐SP coacervates, thus allowing to distinguish between the red‐labelled coacervates from green‐labelled HeLa cells using a custom‐made image analysis pipeline employing the Cell profiler software^[^ ^53^, ^54^ ^]^ (see methods). After 30 minutes of incubation in the presence of inhibitors, the cells were fixed and observed using confocal z‐stack, which enabled segmenting the volume of the cells and the coacervates separately (schematically shown in Figure 5C,D). Coacervates localized in different regions (extracellular space, bound to the outside or the inner cell membrane, or the intracellular space) could then be counted (Figure 5E,F). Cells incubated with SMIFH2 exhibited a significantly lower number of fully internalized coacervates compared to the control, confirming the role of linear actin in the uptake mechanism. In contrast, CK666 did not significantly reduce uptake, in agreement with FACS results. We also observed a significant increase in coacervates bound to the external side of the membrane compared to the control, notably for CK666 (183%) and nocodazole (275%). Notably, nocodazole significantly decreased the internalization of coacervates while increasing the number of coacervates attached to the plasma membrane (Figure 5E). On the corresponding fluorescent images of EGFP‐HeLa cells, most coacervates were confirmed to remain located on the external side of the cell membrane (Figure 5F). This effect of nocodazole is reminiscent of the emerging phenomena of cross‐talking and high‐order interactions between actin and microtubules, which controls diverse intracellular processes, including cytoskeleton remodeling, force generation, cell morphogenesis, and cargo transport.^[^ ^55^, ^56^ ^]^ These results indicate that even if coacervates adhere to the cell membrane, inhibiting actin and tubulin polymerization hinder subsequent internalization.

Overall, the results suggest that unbranched actin filaments play a predominant role in facilitating coacervate uptake, in particular through filopodia‐like structures, whereas branched actin networks and supported surface morphologies make minor contributions. In addition, microtubules significantly contribute to coacervates uptake, which might be related to high‐order association between actin filaments and microtubules.

### 2.6 Modulating Coacervate Adhesion to the Cell Membrane Increases Their Cellular Uptake

**Figure 6:**
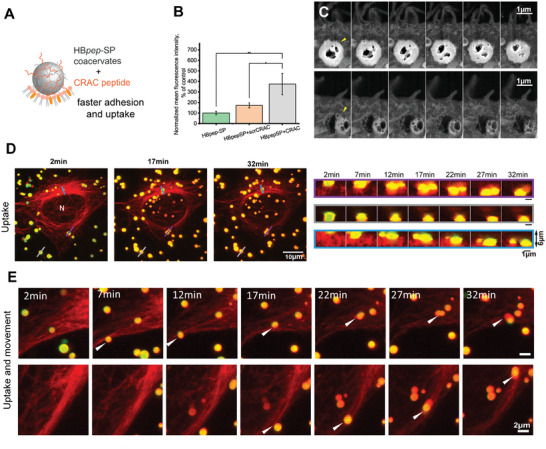
Cellular uptake of HB*pep*‐SP coacervates with the addition of a cholesterol‐binding peptide bearing the CRAC motif. A) Schematic of cholesterol‐mediated adhesion of coacervates with added CRAC peptide. B) FACS analysis of EGFP‐loaded HB*pep*‐SP coacervate with the addition of CRAC peptide and peptide with scrambled CRAC sequence, showing an increase in coacervate uptake only with the correct CRAC sequence. The data were normalized to control (no CRAC peptide added) and shown as a mean ± SD, N = 3. Two sample t‐test was used two compare the groups. * = *P* < 0.05, ** = *P* < 0.01, *** = *P* < 0.001. C) FIB‐SEM *z*‐stack images, showed in series, of cell uptake of HB*pep*‐SP coacervates with the addition of CRAC peptide, showing internalized and dissolving coacervates (yellow arrowheads). Two locations are shown, showing pores in the dissolving coacervates. Top dark area: extracellular space. D,E) Live cell imaging of cellular uptake of HB*pep*‐SP coacervates with the addition of CRAC peptide. Time‐lapse confocal maximum intensity z‐projection images of cells with coacervates (D, left) and close‐up vertical cross‐sections confirming the internalization (D, right). Time‐lapse confocal images of coacervates that underwent internalization and movement inside the cells (E). Red – Spy650‐tubulin, green – EGFP.

We next sought to understand how the recognition and association of coacervates at the plasma membrane facilitates internalization. Since our findings from GUV data indicated that the adhesion of coacervates was cholesterol‐dependent, we hypothesized that adding a cholesterol‐binding molecule within the coacervates would enhance their uptake. We designed coacervates containing recruited cholesterol‐binding peptide (**Figure** **6**A) and examined their uptake and progressive internalization with high‐temporal resolution. We chose a CRAC peptide (with sequence L/VX_(1‐5)_YX_(1‐5)_K/R,^[^ ^57^ ^]^ where X_(1‐5)_ represents a motif containing 1 to 5 of any residues)^[^ ^58^ ^]^ because it has been reported to bind cholesterol with a high affinity, does not disrupt lipid packing, and is not cytotoxic (Figure 6A). The CRAC peptide was added to the stock HB*pep*‐SP in a 1:25 mass ratio before inducing coacervation with EGFP. To verify the presence of CRAC peptide in the coacervates, we recruited CRAC labelled with the red fluorophore TAMRA within HB*pep*‐SP and HB*pep*‐SP‐EGFP loaded coacervates, and visualized its encapsulation by fluorescence microscopy. As shown in Figure S20 (Supporting Information), the TAMRA‐labelled CRAC peptides were uniformly distributed within the coacervates. FACS analysis of cells treated with EGFP‐loaded HB*pep*‐SP coacervates after a 30 min incubation time showed an accelerated uptake with the CRAC peptide. In a control experiment where the CRAC sequence was scrambled (Table S1, Supporting Information), no effect on cell uptake was observed, confirming that cholesterol binding plays a key role in mediating coacervate uptake (Figure 6B).

Furthermore, the CRAC peptide did not affect the uptake of Alexa 488‐transferrin, which is known to enter cells by clathrin‐dependent endocytosis (Figure S21, Supporting Information). These results indicate that cholesterol‐mediated adhesion to the bilayer –which can have an additive effect to specific adhesion caused by ligand‐receptor interactions^[^ ^59^ ^]^– improves coacervate uptake and that enhancing binding to the cell membrane may be an efficient strategy to optimize cell uptake for therapeutic applications. To further confirm the internalization of HB*pep*‐SP coacervates with the added CRAC peptide, we used Focus Ion Beam (FIB)‐SEM (Figure 6C and Movie S1, Supporting Information). FIB‐SEM combines the benefits of TEM and SEM, enabling the visualization of intracellular structure and the acquisition of volume information through serial sectioning. Figure 6C is a FIB‐SEM image of HeLa cells after 3 h of incubation with HB*pep*‐SP coacervates loaded with both CRAC and EGFP. The image shows internalized and partially disassembled coacervates inside the cells.

To dynamically visualize HB*pep*‐SP coacervates with CRAC peptide, we performed live cell imaging in HeLa cells using both actin and tubulin imaging dyes. The experiment with actin dye was inconclusive as the dye was adsorbed by the coacervates, making it challenging to establish any colocalization and enrichment of contact sites in actin (Figure S22, Supporting Information). However, in live cell imaging of HeLa cells stained with tubulin (Figure 6D and Movie S2, Supporting Information), the coacervates displayed a variety of behaviors (Figure 6D,E). Some droplets moved outside the cells (Figure 6D, grey profile), and some underwent slow internalization near the nuclear periphery (Figure 6D, purple and blue profiles), reminiscent of the sinking behavior observed in TEM and SEM. The majority of coacervates underwent fast uptake at the cell periphery. Their movement was observed within the cells, where they appeared to be colocalized with microtubules (Figure 6E), suggesting that the microtubules may act as guiding tracks for intracellular trafficking of coacervates.^[^ ^60^ ^]^

## 3 Conclusion

**Figure 7:**
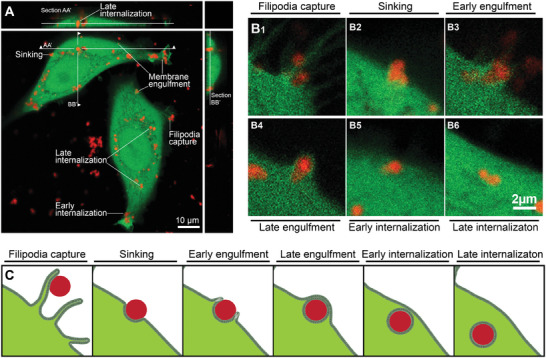
Observed cellular uptake behaviours of HB*pep*‐SP coacervates A) Confocal z‐stack imaging overview showing the uptake of mCherry‐loaded coacervates in HeLa‐GFP, with multiple coacervates detected in the cytoplasm (late internalization) at different stages of uptake in the same image. B) Representative enlarged views of mCherry‐loaded coacervates in different steps of uptake. C) Schematics of different stages of coacervate uptake observed by TEM, confocal fluorescence, and live cell imaging.

The results presented here can be reconciled in terms of a unified mechanistic scenario: the therapeutic carriers, HB*pep* and HB*pep*‐SP coacervates, do not passively cross the membrane barrier. This is established by our GUVs studies, which reveal adhesion of coacervates at the membrane surface with little or no evidence for passive internalization. Instead, the coacervates engage an active, energy‐consuming mechanism. The process begins with the recognition of the coacervate droplets. Our imaging experiments reveal that the coacervates are captured by filopodia‐like protrusions, which decorate the cell surface, in a manner akin to phagocytosis. Our model membrane experiments suggest that this binding and recognition of the coacervates is influenced by the molecular composition: the presence of cholesterol, and thus the increased bending rigidity and binding to the membrane, promote coacervate‐membrane interactions and increase the final uptake efficiency. Next, our HAADF‐STEM and SEM results establish that the ensuing wrapping of the droplet by a gradual deformation and the topological rupture of the plasma membrane (**Figure** **7** and Movie S3, Supporting Information), which sinks and engulfs the endocyte into the cell, is an active process driven by energy‐dissipating cytoskeletal remodeling. This is confirmed in inhibition assays targeting actin and tubulin polymerization, which reveals the essential roles of formin‐mediated actin and tubulin polymerization. Together, the three lines of evidence – the appearance of filopodia‐like membrane protrusions, the critical roles of cytoskeleton remodeling, and topological transition at the membrane surface – suggest an internalization route that shares attributes of two canonical endocytic routes, namely phagocytosis and micropinocytosis driving intracellular delivery of coacervates.

While there are indications that the cell uptake of coacervate microdroplets is a mechanosensitive process – and thus partially regulated by the material properties of the droplets – which mechanoreceptive molecules may be involved in the sensing process is currently unknown. Unveiling these coacervates/mechanosensing molecule interactions may allow to further enhance the cell uptake kinetics by tuning the viscoelastic properties of the coacervates.

## 4 Experimental Section

### Experimental Design

The study aims to shed light on the cellular uptake of peptide coacervates. Using a multimodal imaging approach, a combination of microscopy techniques augmented by cell uptake experiments were employed in the presence of inhibitors targeting the cytoskeleton. A pipeline using 3D confocal imaging was developed, to complete FACS analysis, and distinguish the different steps of internalization, especially in the presence of inhibitors. Additionally, GUVs with various lipid compositions were used to explore the attachment efficiency to model lipid bilayers.

### Peptides

HB*pep* was purchased from GL Biochem (Shanghai) Ltd, China, and subjected to an additional purification step by High Performance Liquid Chromatography (HPLC) using a C8 column. HB*pep*‐SP was synthesized according to the protocol described previously.^[^ ^6^ ^]^ To prepare the coacervates, 10 mg mL^−1^ of HB*pep* or HB*pep*‐SP in 10 mM acetic acid was pipetted into phosphate buffer (pH 6.5, 100 mM ionic strength) at a volume ratio of 1:9 as previously described.^[^ ^6^ ^]^ CRAC and CRAC scrambled peptides were synthesized in a solid‐phase peptide synthesizer (CEM Liberty Blue) using general protocols. First, Wang resin, which supports the growth of peptide, was swollen in N,N′‐dimethylformamide (DMF) for 30 mins before being transferred into the reaction vessel. Fluorenylmethyloxycarbonyl (Fmoc) protected peptides, activator N,N′‐dimethylcarboimide (DIC), activator base ethyl cyanohydroxyiminoacetate (oxyma), and piperidine were dissolved in DMF, and transferred into the synthesizer. Peptide and piperidine were injected into a heated reaction vessel to remove Fmoc functional group. Then DIC and oxyma were injected, and heat was applied to the reaction vessel to couple the deprotected amino acid with peptide chain. The two steps repeated until all amino acids were connected. After synthesis, the resin was washed by dichloromethane (DCM) and DMF. Then a cocktail solution containing 95% trifluoric acid (TFA), 2.5% triisopropylsilane (TIPS), and 2.5% H2O was added into the resin to cleave the peptides from the resin. The resin was soaked in the cocktail for 2 hours, and cold diethyl ether was added to precipitate peptides. The precipitates were collected after centrifugation. After drying in nitrogen, they were dissolved in 5% acetic acid and purified by high‐performance liquid chromatography (HPLC). The products were isolated by lyophilization.

### DLS Zeta‐Potential Measurements

For zeta‐potential measurement, 100 ul HBpep coacervate solution loaded with and without EGFP was added to 900 ul PBS and loaded into a disposable folded capillary cell. The measurements were performed on Zetasizer Nano ZS (Malvern, UK), in automatic mode.

### Giant Unilamellar Vesicles (GUVs) Preparation for Studies of Coacervate Attachment with Varying Lipid Charge and Cholesterol

GUVs were prepared via gel‐assisted formation on PVA. Briefly, 5% or 10% (w/w) solution of Polyvinyl alcohol (PVA) was prepared by stirring PVA in water at 90 °C. 100 µL of PVA solution was added onto an ozone‐cleaned microscope coverslip, which was then dried in an oven at 50 °C for 45 min. 25 µL of lipids (1 mg mL^−1^) was subsequently spread onto the dried PVA film and placed under vacuum for 30 min until the solvent evaporated. 350 µL of sucrose solution (187 mM) was added to the PVA film and incubated for up to 2 hours to allow GUV formation. The GUVs were then pipetted into an Eppendorf tube and stored at 4 °C until further use.

For HB*pep* coacervates‐GUV interaction studies, 5 µL of the GUV solution was added to 200 µL of phosphate buffer and incubated for 5 min at room temperature. 20 µL of EGFP‐loaded coacervates was then added to the GUVs for interaction studies. Fluorescence microscopy images were acquired using a Delta vision elite inverted epifluorescence microscope with Olympus IX‐71 base fitted with 10×/0.40, 20×/0.75, 40×/0.65‐1.35 oil objectives (Olympus, Tokyo, Japan), DAPI, TRITC and FITC Semrock filters (New York, NY), a mercury lamp (Intensilight C‐HGFIE, Nikon Corporation, Tokyo, Japan), and a high‐precision motorized stage. Images were collected using Softworx 4.1.0 (Applied Precision, Inc., Issaquah, WA) and processed using ImageJ. The number of attached coacervates were count and divided by the perimeter of the individual GUV. 8–10 GUVs were analyzed for each group For HB*pep*‐SP coacervates, 10 µL of GUV solution was added to 140 µL of phosphate buffer followed by 10 µL of HB*pep*‐SP coacervates. To better visualize the effective contact of GUVs with coacervates and validate the data obtained by fluorescence microscopy, the images were acquired using a high‐speed spinning‐disc confocal microscope (Nikon Ti2‐E) equipped with a 100 × 1.45NA Plan‐Apo objective lens and an ORCA‐Fusion sCMOS camera (Hamamatsu Photonics). For the quantification, 8 fields of view were used for each group. All GUVs were selected, and the total perimeter was calculated. The total number of attachment of coacervates per GUV unit length was quantified by counting all attached coacervates and then dividing this number by the total GUV perimeter length (summarized in Figure S23 and Table S2, Supporting Information). For the purpose of ensuring consistency and comparability across different experiments, the data were normalized against the baseline attachment of coacervates to GUV containing solely POPC and plotted as relative increase expressed as fold change.

### Cell Culture

HeLa cells, HepG2 cells (ATCC, USA) and HeLa‐GFP cells (Cell Biolabs, INC.) were cultured on DMEM (Gibco) and EMEM (ATCC) media supplemented with 10% FBS and penicillin/streptomycin solution (Gibco) in a humidified atmosphere at 37C° and 5% CO_2_. Cells were routinely tested for mycoplasma using Mycostrip kit (Invivogen).

### Coacervate Preparation for Imaging and Cell Uptake Experiments

For cell uptake experiments, reduced serum Optimem media (Gibco) was used. Coacervates were formed by mixing one part of peptide stock solution in 10 mM acetic acid (10 mg ml^−1^ for HB*pep*‐SP or 10 or 20 mg ml^−1^ for HB*pep*) with 9 parts of 10 mM sodium phosphate buffer with 100 mM sodium chloride, pH 7.5 for HB*pep* and 6.5 for HB*pep*‐SP containing EGFP, mCherry in a 0.1 mg ml^−1^ or EGFP in 0.05 and recombinant ferritin in 0.01 mg ml^−1^ concentration. The media in cell culture dishes or flasks were replaced with Optimem, and coacervate mixtures were gently pipetted in. For HB*pep*, the final peptide concentration in Optimem was 0.2 or 0.4 mg ml^−1^, for HB*pep*‐SP – 0.1 mg ml^−1^. To prepare the coacervates with CRAC or TAMRA‐CRAC labelled peptide, 10 mg mL^−1^ HB*pep*‐SP mixed with CRAC (1:25 mass ratio) in 10 mM acetic acid was pipetted into phosphate buffer (6.5 pH, 100 mM ionic strength) containing EGFP at a volume ratio of 1:9.

### Live Cell Imaging of HBpep Coacervates Uptake in HeLa‐GFP Expressing Cells

HeLa GFP‐expressing cells of 2 × 10^5^ were seeded in 35 mm glass bottom dishes (Mattel, USA) and cultured for 24 h. Then the cell culture media was replaced with 800 µl of Optimem (Gibco) and put in the observation chamber of the confocal microscope (LSM980, Zeiss) with a x63/1.40 Oil DIC objective (420 782, Zeiss). mCherry‐loaded HBpep coacervates (final HB*pep* concentration 0.2 mg ml2 × 10^5^) were added to the dish. Z‐stack images were collected across 11 µm with 1 um slices. GFP and mCherry fluorescence channels were used. Images were processed using Zen blue edition (Zeiss, Germany) and Image J (NIH, USA) software.

### Live Cell Imaging of HBpep‐SP Coacervate Uptake in Hela Cells

HeLa cells of 3 × 10^5^ were seeded in 35 mm glass bottom dishes (Mattel, USA) and cultured for 24 h. For membrane engulfment studies, the cells were stained with CellMask Deep Red plasma membrane stain (Thermofisher scientific, USA) for 1 min, then rinsed three times with PBS. For cytoskeleton staining, cells were stained with 1 µl Spy555‐actin or Spy650‐tubulin (Spirochrome, Germany) in 1 ml of cell culture media for 2 h and rinsed 4 times with PBS. The PBS was replaced with live cell imaging solution (Thermofisher scientific, USA), and the dishes were mounted in the observation chamber with temperature and CO_2_ control. HB*pep*‐SP coacervates were loaded to the dishes and kept for 2 min before imaging until coacervates settled on the cell surfaces. Time‐lapse Z‐stack images were then collected with 5‐min time interval for 20 or 30 min across the cell with 0.3 um slices, using high‐speed spinning‐disc confocal microscope (Nikon Ti2‐E) equipped with a 100 × 1.45NA Plan‐Apo objective lens and an ORCA‐Fusion sCMOS camera (Hamamatsu Photonics). GFP, Cy5, and mCherry fluorescence channels were used. Images were processed using Image J (NIH, USA) software.

### FRAP Analysis

Fluorescence recovery after photobleaching (FRAP) experiments were performed on a spinning disc confocal (SDC) system built around a Nikon Ti2 inverted microscope equipped with a Yokogawa CSU‐W1 confocal spinning head and a 100×/1.4NA oil immersion objective. Samples were placed in a 96‐well microplate (Ibidi). The experiments were conducted either immediately or one hour after pipetting coacervates in the Optimem media at room temperature. Typically, images were acquired with an interval of 20 / 40 s between subsequent images. Before photobleaching, 3 to 5 images were recorded.

To analyse FRAP experiments, ROIs were manually selected to measure the average intensity of the bleached area (I_B_), the non‐bleached area (I_REF_), and the background of the image (I_BG_) in each frame. These intensity values were used to calculate FRAP curves for the bleached half, according to:

(1) $$FRAP\left(t\right)=\frac{I_{B}\left(t\right)-I_{BG}\left(t\right)}{I_{REF}\left(t\right)-I_{BG}\left(t\right)}$$

The above fraction was then normalized to the recovered percentage according to the pre‐bleaching frames (100%) and the first post‐bleaching frame (0%) in GraphPad Prism. HB*pep*‐coacervates loaded with two EGFP concentrations (0.1 and 0.01 mg ml^−1^ in the coacervate mixture) or DAPI (0.25 mg ml^−1^) were added to Optimem in 1:4 ratio, and HB*pep*‐SP were loaded with 0.1 mg ml^−1^ EGFP and added to Optimem in 1:9 ratio.

### FACS Analysis of Cell Uptake in the Presence of Chemical Inhibitors Targeting Cytoskeleton

Hela cells or Hela‐GFP expressing cells were seeded in 24 well plates (0.5 × 10^5^ cells/well) and grown for 24 h. For the analysis, the media was substituted with Optimem with endocytosis inhibitors (5 mM mβCD, 10 µg ml^−1^ nocodazole, 20 µM SMIFH2, 100 µM of CK666, or 100 µM EIPA) and incubated for 30 min (50 min with mβCD). Then, HB*pep*‐SP coacervates loaded with mCherry were added (the final peptide concentration in Optimem for HB*pep*‐SP was 0.1 mg ml^−1^) and incubated for 30 min. Cells were washed trice with cold PBS to remove the non‐internalized coacervates, detached with Accutase (Invitrogen, USA), washed with FACS buffer (PBS containing 2% FBS), resuspended in 350 µl FACS buffer. FACS analysis was performed on BD LSRFortessa X‐20 Cell Analyzer (BD Biosciences, USA). The data were processed in Flojo software (Flojo, USA).

### Coacervate Cellular Uptake Analysis Using Cell Profiler with Customized Pipeline

To complement the FACS data, the HeLa‐GFP expressing cells were grown in 35 mm glass bottom dishes (Mattek, USA) for 24 hours till 50–60% confluency. Cells were treated with inhibitors for 30 min, incubated with HB*pep*‐SP mCherry‐loaded coacervates for 30 min, fixed and imaged. Each condition were imaged in 7–8 different spots each by confocal microscopy to obtain 3D stacks (Movie S3, Supporting Information). A minimum of 250 average coacervates per spot were analyzed and up to an average of 640 coacervates per spot, (Table S3, Supporting Information). Each spot was preprocessed using Python to be used in CellProfiler for analysis. The CellProfiler pipeline include an intensity rescale, median filter, thresholding (three classes Otsu) and segmentation of the cells and coacervates respectively (using a watershed algorithm). The green channel was used to segment the cell 3D volume (GFP‐expressing cell cytoplasm). The identified cell volume was dilated by 1 µm to obtain the membrane external side and remaining extracellular space (1 µm dimension chosen to be as the mean diameter of a single coacervate). The identified cell volume was contracted by 1 µm to obtain the membrane internal side and remaining intracellular space. The red channel was used to segment the individual mCherry‐loaded coacervates. Analyzing z‐stack images, each coacervate was identified as belonging to the intracellular, internal cell membrane, external cell membrane or extracellular volumes and resulting table was exported in a comma‐separated value (csv) file. A postprocessing using python counted the coacervates in the different regions. Counts were first normalized for each sample, dividing the number of coacervates in each region by the total number of coacervates present (Figure S19, Supporting Information). For inhibitor studies, the counts were further normalized, dividing the values in each region by the control values (without inhibitor). T‐tests and plotting were performed in Excel and OriginLab2023b. The obtained normalized values could therefore be compared to FACS data (Figure 5A,B).

### TEM

Cells were grown in Mattek dishes (Mattek) or T25 flasks (Corning) till ≈60% confluence. Then the media was substituted with Optimem (Gibco) containing coacervates loaded with EGFP or ferritin (HB*pep*‐SP peptide) or EGFP and ferritin (HB*pep*) as model cargos and placed in the incubator for 15 min or 3 h incubation followed by washing with PBS and fixation with 2.5% glutaraldehyde (EMS) in 0.1 M phosphate or cacodylate buffer, pH 7.4. Cells were postfixed with 1% OsO_4_ solution with added 2% potassium ferricyanide for 1.5 h on ice. Cells grown in Mattek dishes (HeLa transfected with HB*pep* coacervates) were embedded as monolayers, while cells in T25 flasks. Other coacervates and cell lines were scraped after fixation and embedded as pellets. Cells in Mattek dishes were additionally stained with 1% of uranium acetate solution overnight in the dark. All samples were further subjected to dehydration through graded ethanol series and embedded in Durcupan resin. Ultrathin sections (70 or 100 nm) were obtained using Leica FC6 microtome. Imaging was performed on a JEM‐2100F electron microscope operated at 200 kV in HAADF‐STEM mode. The images were processed in Digital Micrograph (Gatan, USA), and Fiji (NIH, USA).

### SEM

HeLa and HepG2 cells were cultured on glass coverslips in 6 well plates. Cells were incubated with EGFP‐loaded coacervates followed by 15 min incubation at 37 °C in the incubator, washed with PBS, fixed with 2.5% glutaraldehyde in PBS for 30 min at room temperature, and dehydrated in a graded ethanol series. Samples were CO_2_ critical point dried (Samdri‐PVT‐3D, Tousimis) and coated by sputtering with a 10 nm platinum layer. Imaging was performed on a field emission scanning electron microscope (SEI mode, 10–15 keV, JSM‐7600F, JEOL).

### AFM

Topographical images and force spectroscopic measurements of the peptide samples were taken using a commercial AFM system equipped with a liquid droplet holder (Cypher S, Oxford Instruments, U.K.). The samples were prepared by first forming a droplet of 40 µL buffer on mica. Then 10 µL HBpep‐SP peptide coacervates loaded with EGFP were added to the buffer droplet. After 10 minutes of incubation, the sample was rinsed with buffer before placing in the AFM chamber. All measurements were carried out at room temperature. The AC mode was employed to image the peptides for minimal tip‐induced deformation. To gather force‐separation curves, force spectroscopic mapping was performed on the same scan area as the topographic image. Young's moduli were obtained by fitting the force profiles, taken on the peptide particle, with the Hertzian model for a conical indenter.^[^ ^61^, ^62^ ^]^ The reported value was the averaged value from all recorded force profiles. The spring constants of the AFM probes (BioLever mini, Oxford Instruments, eStore Asia; nominal spring constant: 90 pN nm^−1^) were calibrated using the thermal method.^[^ ^63^ ^]^

The Hertzian model for a conical indenter was given by the equation:

(2) $$F=\frac{2E\tan\theta }{\pi \left(1-\upsilon^{2}\right)}\;\delta^{2}$$

where, *F*, δ, θ, *E*, υ, are the force, indentation, half‐cone angle of the indenter, Young's modulus of the sample, and Poisson ratio of the sample, respectively.

### FIB‐SEM

Cells were grown in Mattek dishes till ≈80% confluence. Then the media was substituted with Optimem (Gibco) containing coacervates loaded with EGFP (HB*pep*‐SP peptide with addition of CRAC peptide) for 3 h incubation followed by washing with PBS and fixation with 2.5% glutaraldehyde (EMS) in 0.1 M cacodylate buffer, pH 7.4. Cells were postfixed with 1% OsO_4_ solution with added 2% potassium ferricyanide, treated with 1% thiocarbohydrazide, subjected to another round of 1% OsO_4_ staining, and additionally stained with 1% of uranium acetate solution overnight in the dark. All samples were further subjected to dehydration through graded ethanol series and embedding in Durcupan resin. Small pieces were sawed using jeweler saw, mounted on SEM stabs using carbon paste, sputter‐coated with platinum and imaged in Zeiss Crossbeam 540 microscope (Zeiss, Germany). The parameters used for ion beam milling were: acceleration voltage 30 kV, milling current 300 or 700 pA, section thickness 50 or 73.5 nm. The obtained images were processed in Fiji (NIH, USA).

### Statistical Analysis

Statistics were computed using Origin Pro 2023b. Standard two‐sample Student's t‐test was performed to test for statistical differences between two groups. For comparison of more than two groups, one‐way ANOVA was applied. *P* < 0.05, *P* < 0.01, *P* < 0.001 denoted on the figures as *, **, and *** respectively.

## Author Contributions

A.M., A.S., Q.M.P., Y.M., A.L. performed conceptualization. A.S.,Q.M.P.,K.Z.,Z.W.L.,Y.S.,C.H.,S.L.,R.S.,E.A.M.,S.L.,J.Y. performed investigation. Q.M.P. performed software. A.M. performed supervision. A.S., Q.M.P., S.G., A.M. wrote the—original draft. A.S., Q.M.P., A.M., Y.M., A.P., A.L. wrote—review and edited the original draft.

## Conflict of Interest

The authors AM and YS have filed a US patent on HB*pep*‐SP used in this study.

## Supporting information

Supporting Information

Supplementary Movie S1

Supplementary Movie S2

Supplementary Movie S3

## Data Availability

The data that support the findings of this study are available from the corresponding author upon reasonable request.
