# Supplementary material for: Cellular Uptake of Phase‐Separating Peptide Coacervates
Source: Adv Sci (Weinh). 2024 Aug 30;11(42):2402652. doi: 10.1002/advs.202402652 (PMC11558145; doi:10.1002/advs.202402652)
Supplement: Supplementary file 1 — Supporting Information [file ADVS-11-2402652-s001.pdf]

ADVANCED SCIENCE
Open Access

Supporting Information

for *Adv. Sci.*, DOI 10.1002/advs.202402652

Cellular Uptake of Phase-Separating Peptide Coacervates

*Anastasia Shebanova, Quentin Moana Perrin, Kexin Zhu, Sushanth Gudlur, Zilin Chen, Yue Sun, Congxi Huang, Zhi Wei Lim, Evan Angelo Mondarte, Ruoxuan Sun, Sierin Lim, Jing Yu, Yansong Miao, Atul N. Parikh, Alexander Ludwig and Ali Miserez**

Supporting Information for

Cellular Uptake of Phase-Separating Peptide Coacervates

Anastasia Shebanova *et al.*

*Corresponding author. Email: ali.miserez@ntu.edu.sg

This PDF file includes:

Figuress. S1 to S23
Tables S1 to S3

Other Supplementary Materials for this manuscript include the following:

Movies S1 to S3

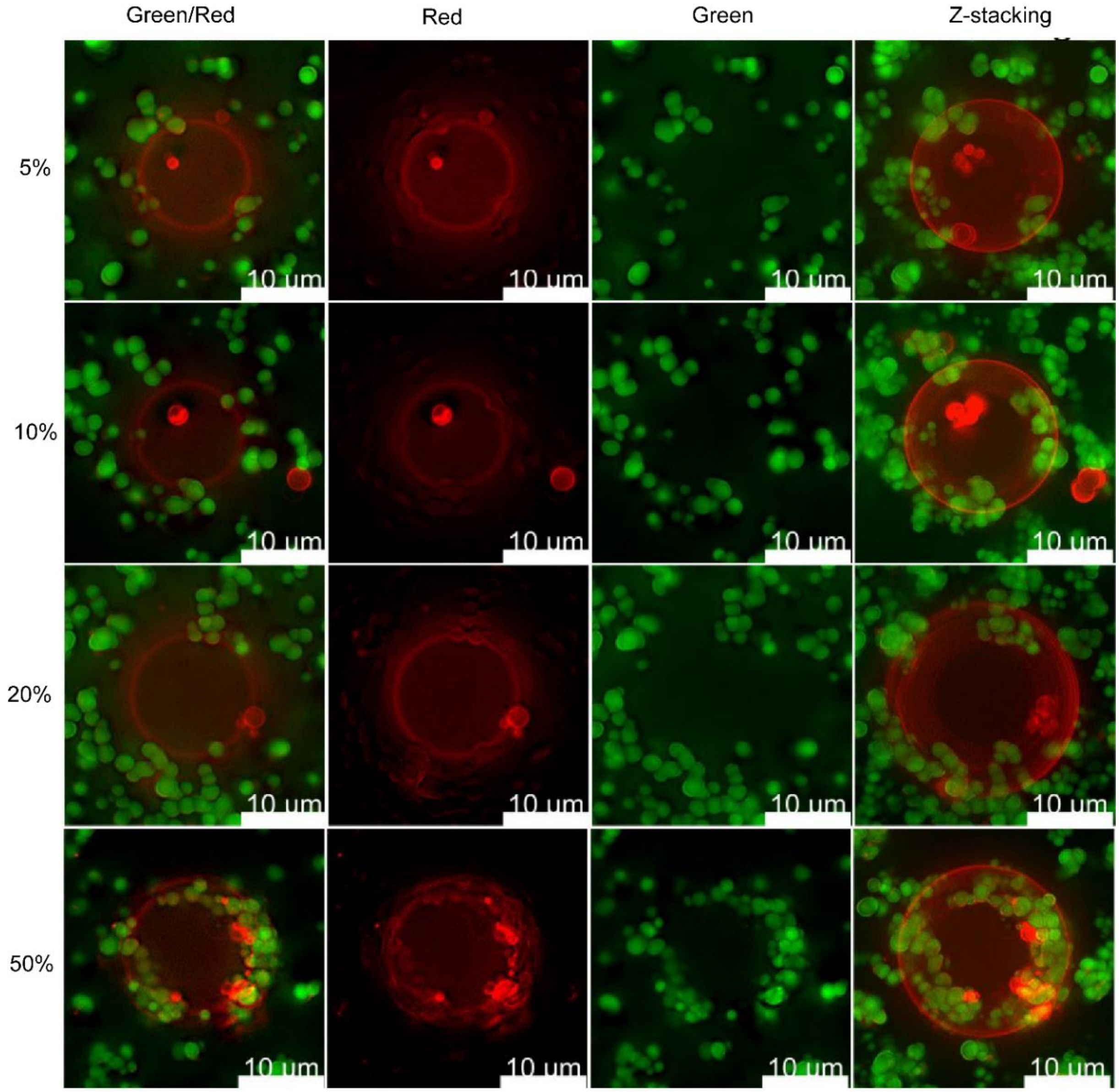

Figure S1. Effect of negatively charged membrane lipids on HB*pep* coacervate attachment. Fluorescence microscopy images of POPC GUVs (Red) with varying levels of POPG is mixed with HB*pep* coacervates entrapping EGFP (green). Representative merged images (left column) of the green and red channel (middle columns) are shown and the right-most column represents a representative z-stack composite of optical sections collected 1 µm apart.

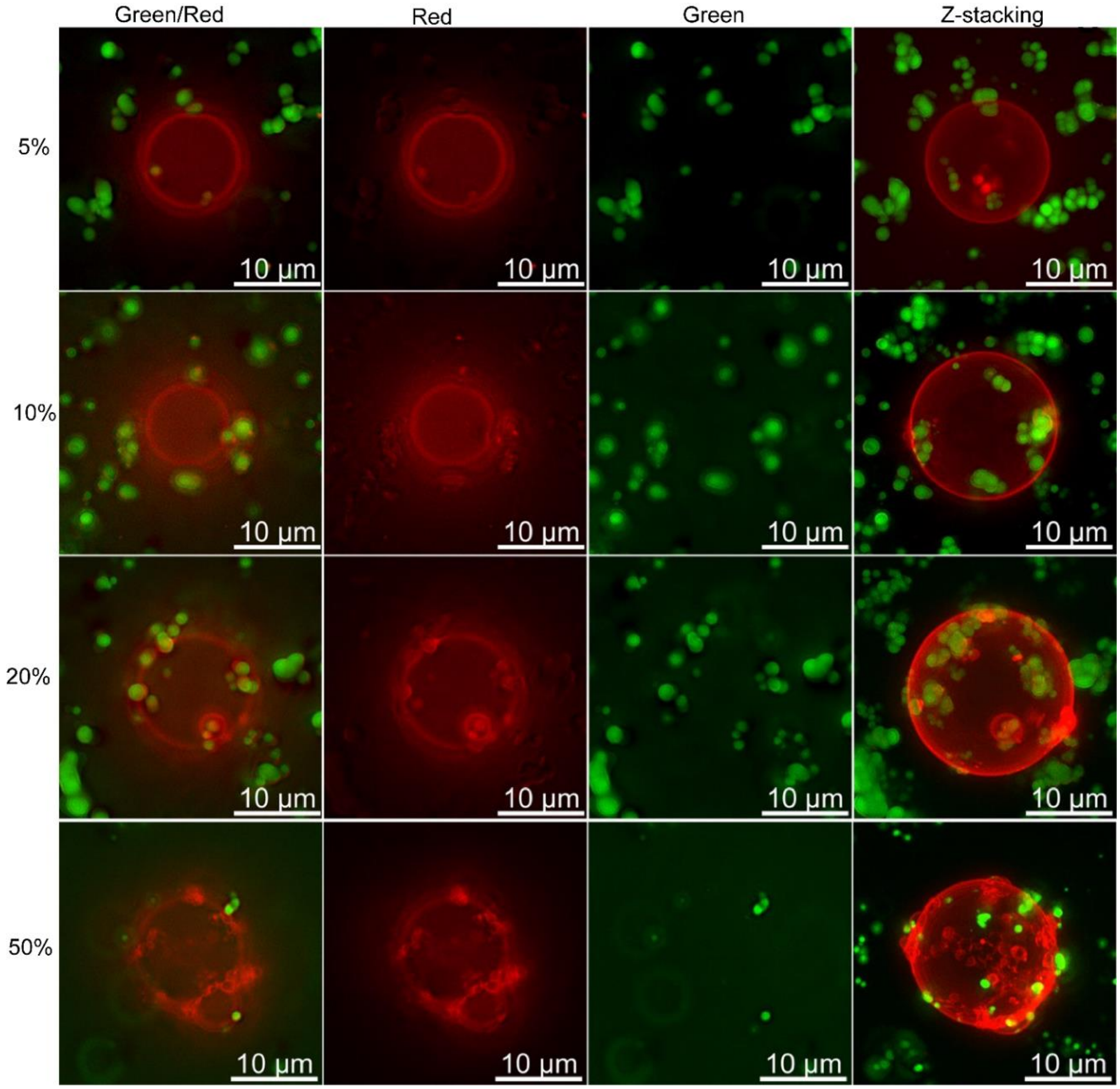

Figure S2. Effect of positively charged membrane lipids on HB*pep* coacervate attachment. Fluorescence microscopy images of POPC GUVs (Red) with varying levels of DOEPC is mixed with HB*pep* coacervates entrapping EGFP (green). Representative merged images (left column) of the green and red channel (middle columns) are shown and the right-most column represents a representative z-stack composite of optical sections collected 1 µm apart.

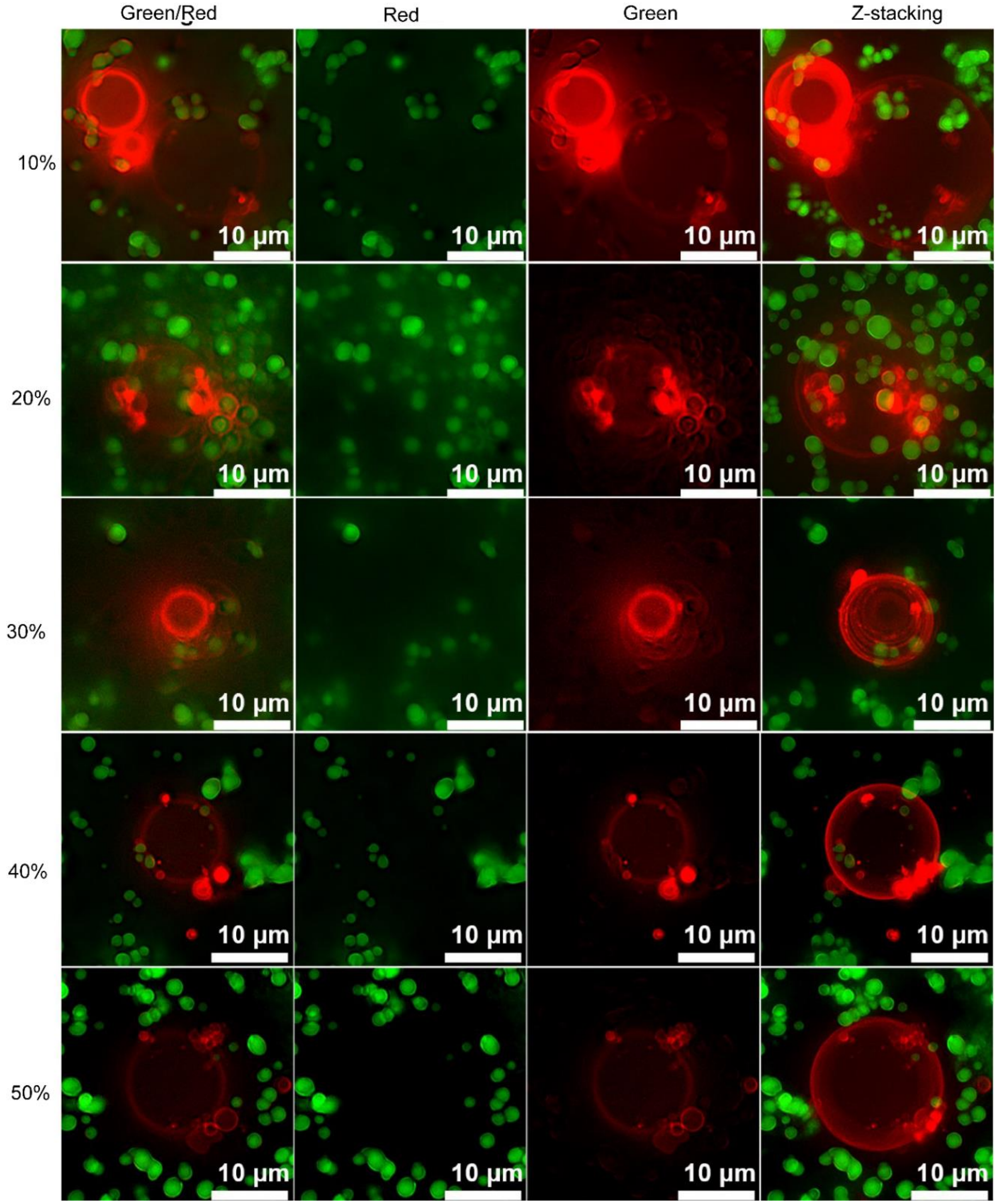

Figure S3. Effect of cholesterol on HB*pep* coacervate attachment. Fluorescence microscopy images of POPC GUVs (Red) with varying levels of cholesterol is mixed with HB*pep* coacervates entrapping EGFP (green). Representative merged images (left column) of the green and red channel (middle columns) are shown and the right-most column represents a representative z-stack composite of optical sections collected 1 μm apart.

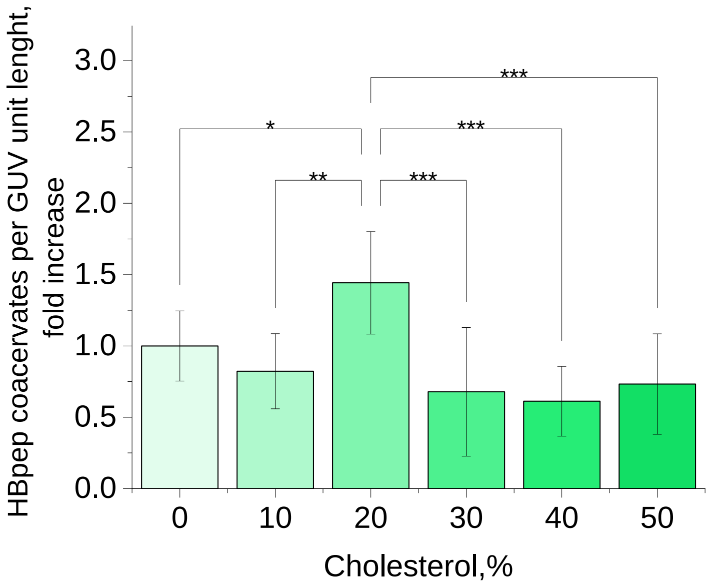

Figure. S4. Plot of attachment of HB*pep* coacervates to POPC GUV with various concentrations of cholesterol. Coacervate attachment is the highest for POPC GUVs with 20% cholesterol. Data are presented as the mean ± SD, N = 10. F. One-way ANOVA was used to compare the groups, * = $P < 0.05$, ** = $P < 0.01$, *** = $P < 0.001$.

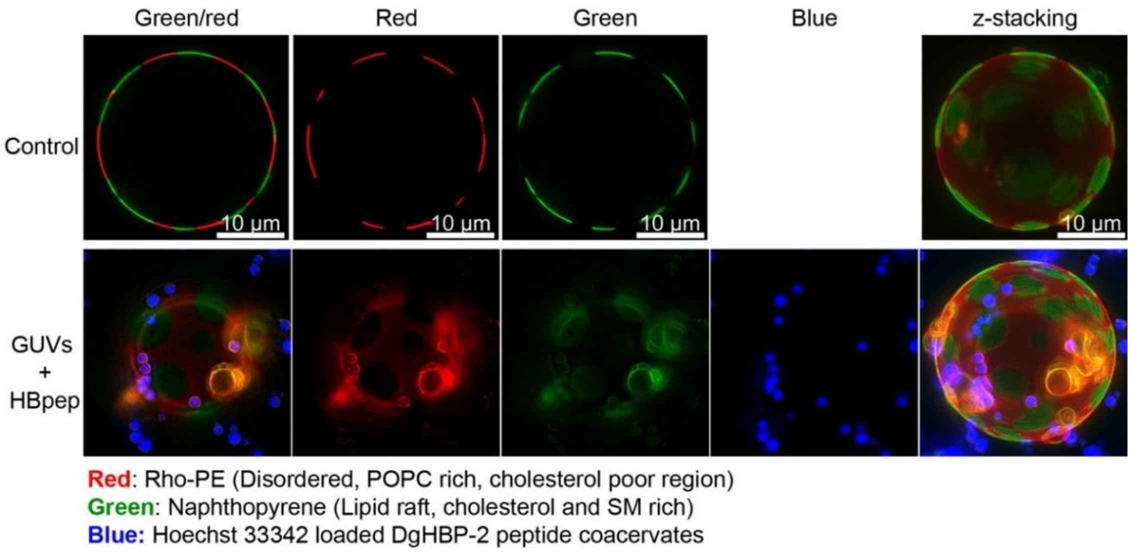

Figure S5. Interaction of HB*pep* coacervates with GUVs mimicking lipid rafts (phase separated liquid ordered and liquid disordered regions). Hoechst 33342-loaded HB*pep* coacervates mixed with GUVs prepared from 40% POPC/40% sphingomyelin (SM)/20% cholesterol in phosphate buffer. Top row (control): disordered phase (Rho-PE, red) and ordered phase (naphthopyrene, green) within a GUV. Bottom row: Attachment of Hoechst 33342-loaded coacervates with GUV containing lipid rafts.

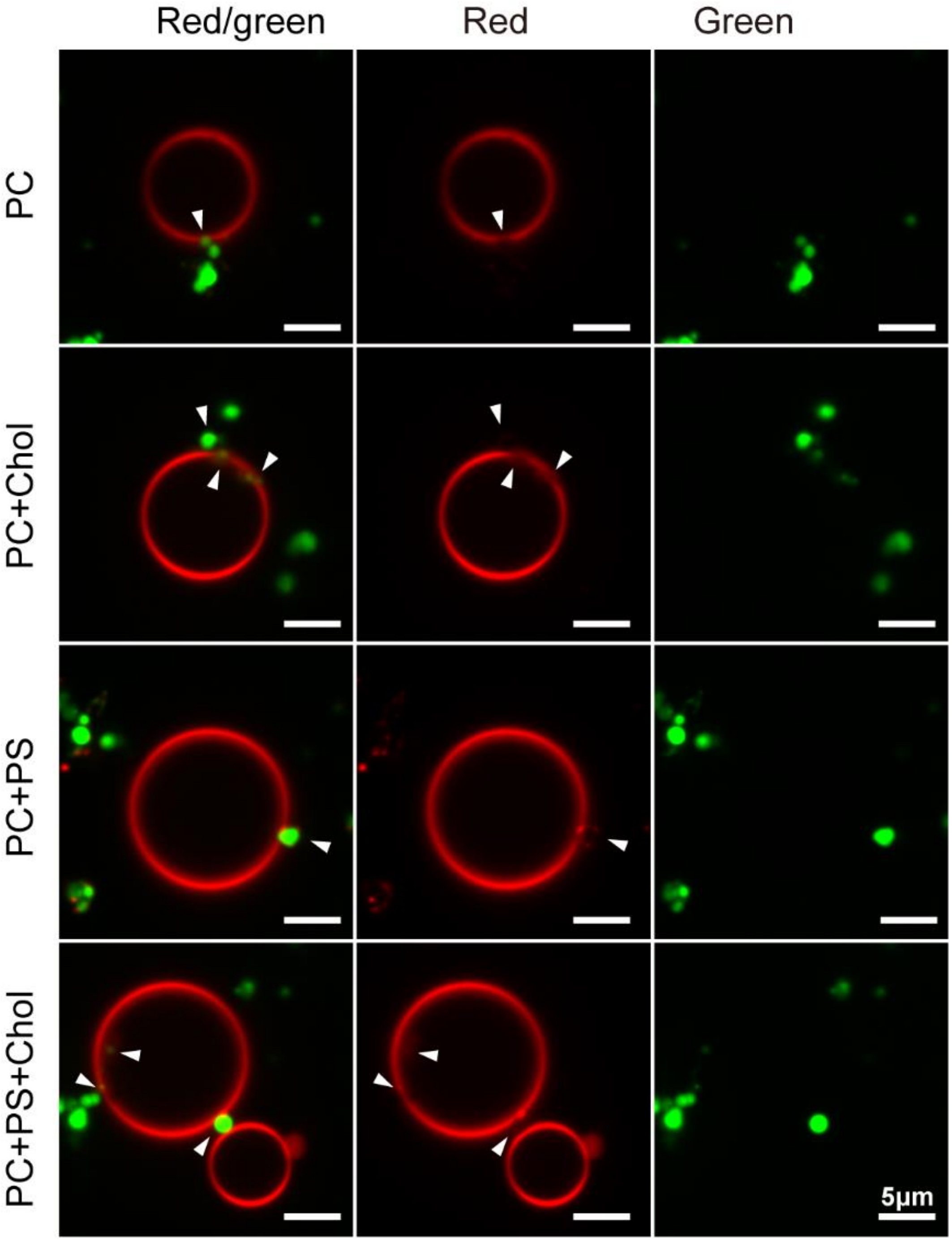

Figure S6. Effect of 20% cholesterol and 10% POPS on HB*pep*-SP coacervate attachment. Confocal microscopy images of POPC GUVs (Red) with/without 20% cholesterol and 10% POPS is mixed with HB*pep*-SP coacervates entrapping EGFP (green). Representative merged images (left column) of the green and red channel (middle and right columns) are shown.

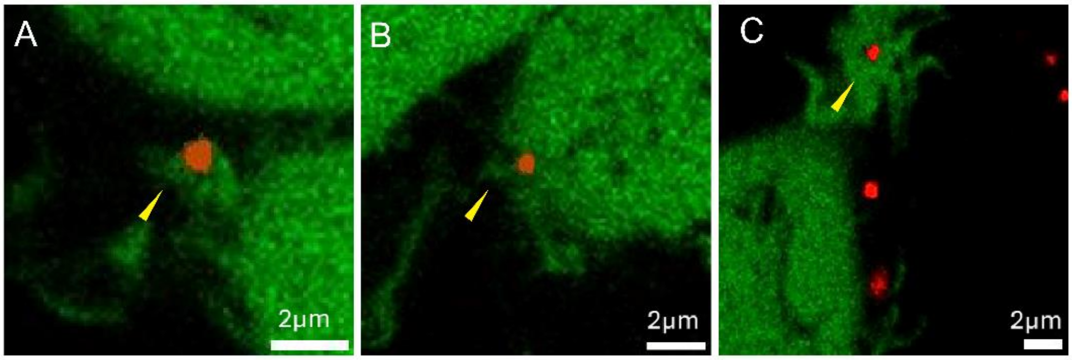

Figure S7. Live cell confocal representative imaging of HB*pep* coacervates loaded with mCherry in HeLa cells expressing GFP. Capture of coacervates (red) filopodia protrusions is indicated with yellow arrows.

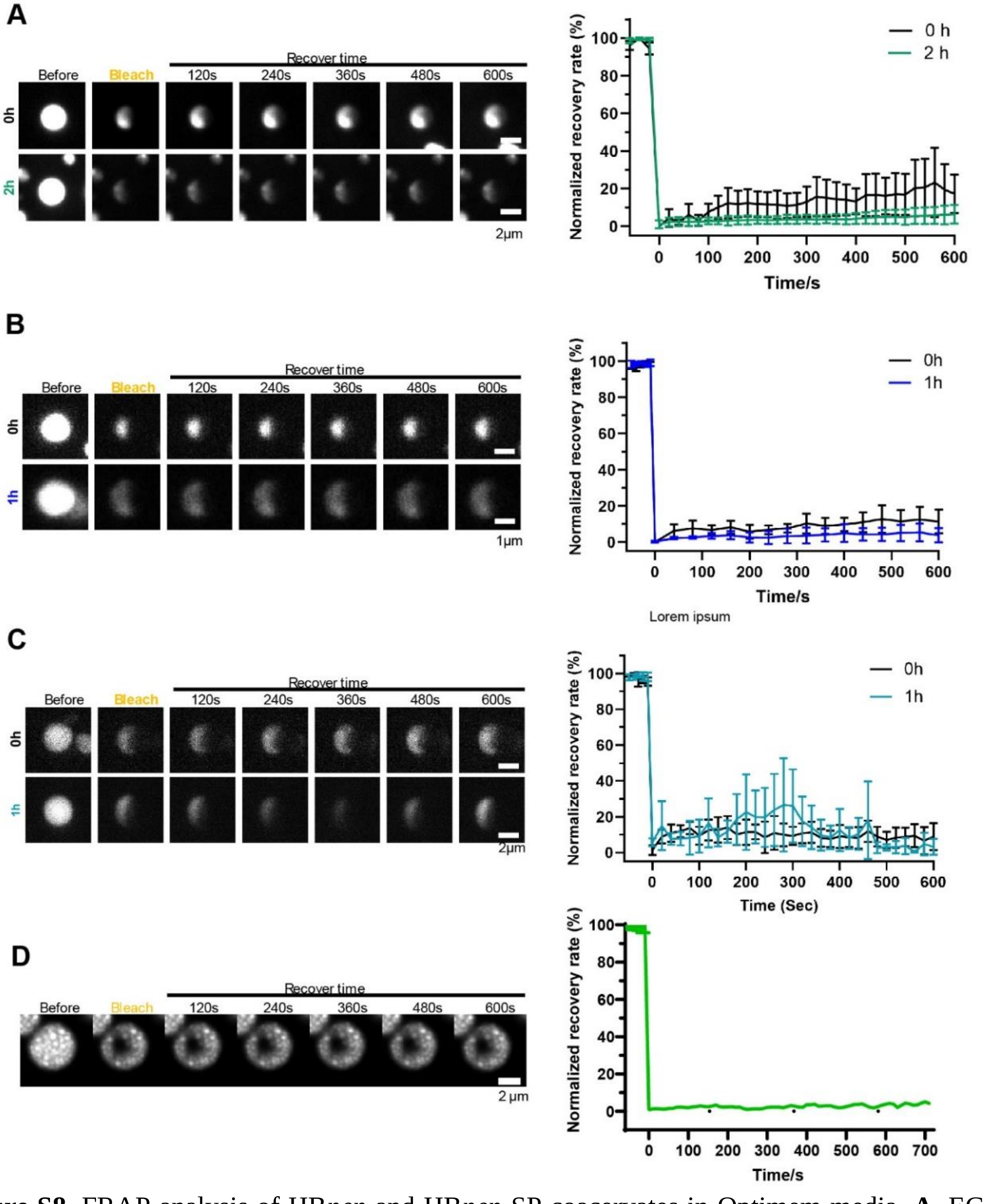

Figure S8. FRAP analysis of HB*pep* and HB*pep*-SP coacervates in Optimem media. **A**. EGFP-loaded HB*pep* coacervates, final concentration of EGFP after addition of the coacervates to Optimem 0.02 mg/ml. **B.** EGFP-loaded HB*pep*, final concentration of EGFP 0.002 mg/ml. **C.** DAPI-loaded HB*pep*, final concentration 0.02 mg/ml. Only 20% of recovery is observed immediately after addition of the coacervates to Optimem indicating the gel-like state of coacervates, and no recovery after 1h. **D**. EGFP-loaded HB*pep*-SP, final concentration of EGFP 0.01 mg/ml in Optimem. No recovery is observed upon addition of coacervates to Optimem.

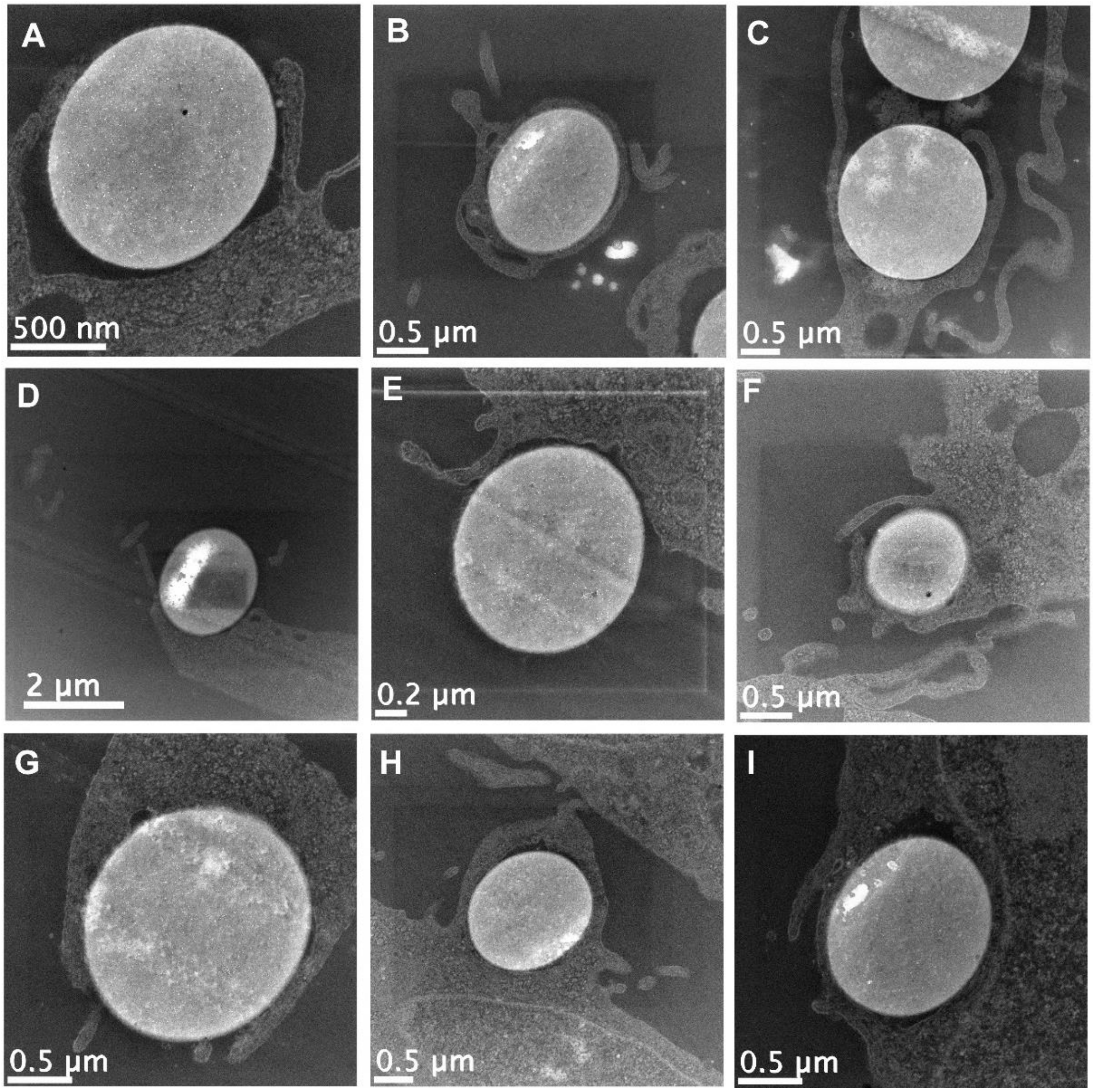

Figure S9. HAADF-STEM images of ferritin-loaded HB*pep*-SP coacervates uptake of HeLa cells after 15 min of incubation with coacervates demonstrating initial stages of the uptake process. **A-C,F.** Filopodia capture and progressive membrane engulfment. **D, E, G-I** Cup formation can be observed**.**

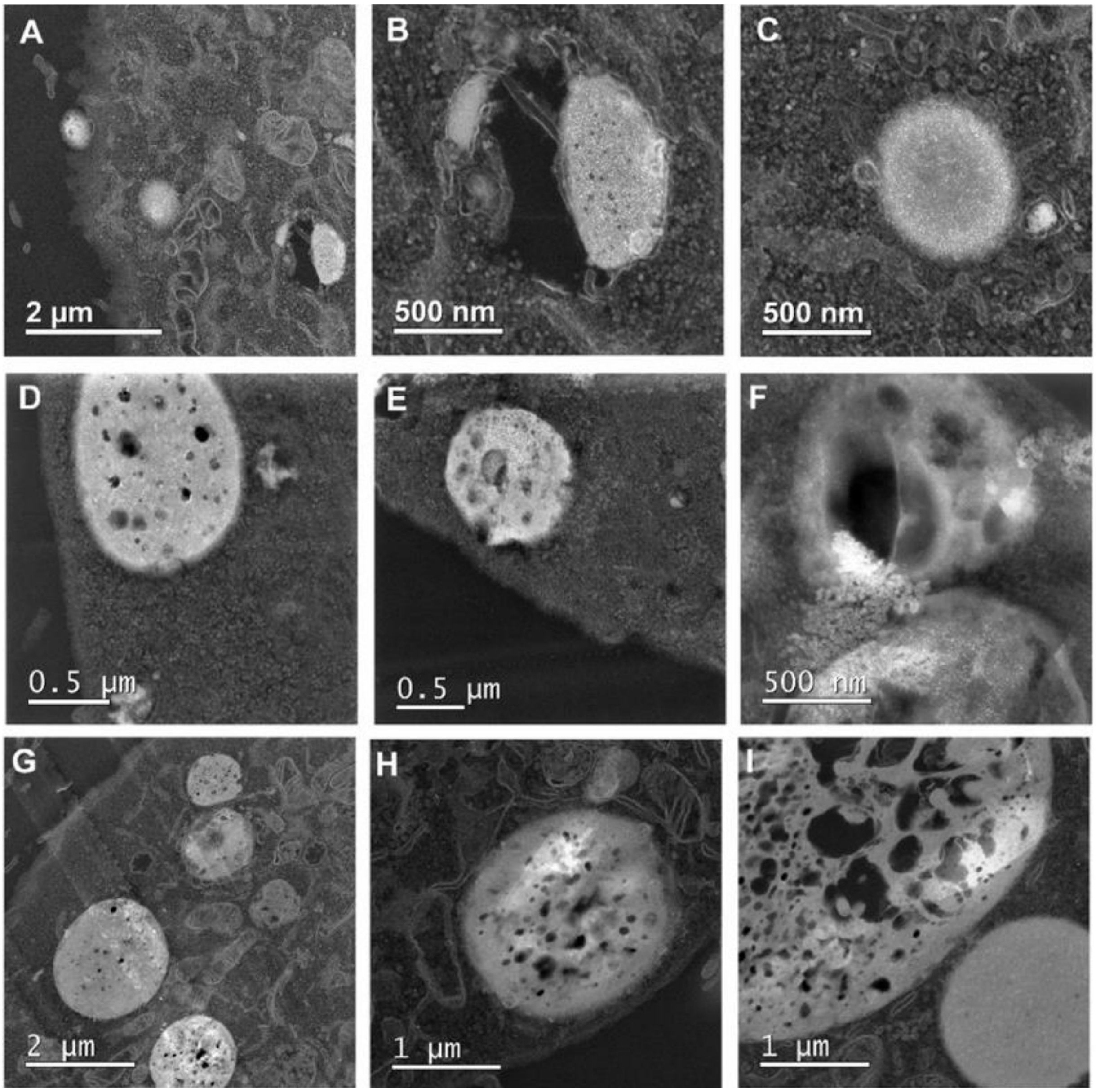

Figure S10. HAADF-STEM images of HB*pep* (**A-C)** and HB*pep*-SP (**D-I**) coacervates loaded with different cargos (A-C with both ferritin and EGFP, D-F with ferritin and G-I with EGFP) after 3 h of incubation in HeLa cells. More pronounced coacervate disassembly is seen in the case of HB*pep*-SP coacervates, as evidenced by their extensive porous structure.

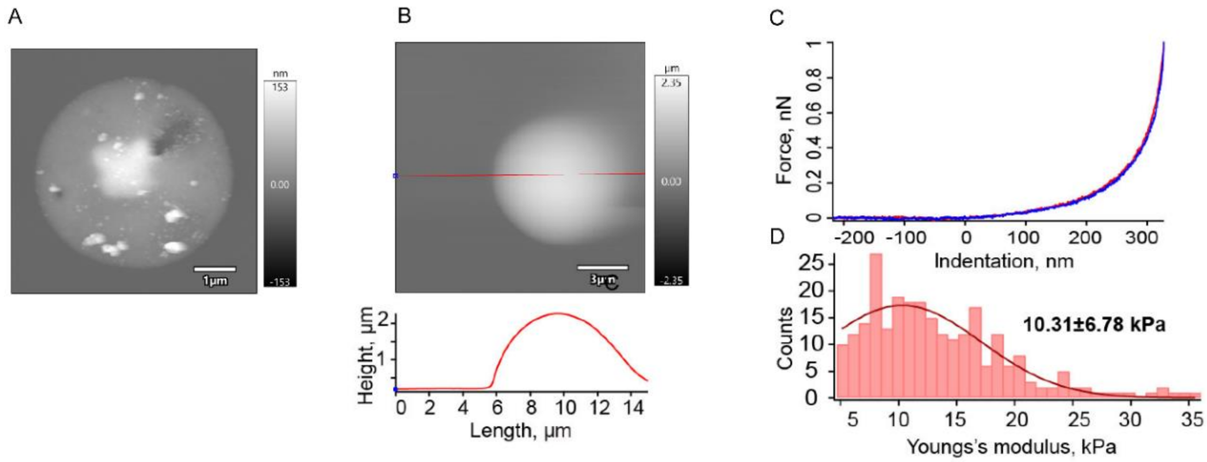

Figure S11. Nanoindentation of HB*pep* and HB*pep*-SP coacervates by AFM. **A**. Representative image of an HB*pep* coacervate spreading on the mica. **B-D**. AFM nanoindentation measurements in liquid of the HB*pep*-SP coacervates. AFM surface topography image of a coacervate with the height profile (B). Force extension curves (C) and distribution of Young's modulus of the coacervate (D) showing that the Young's modulus is in the kPa range.

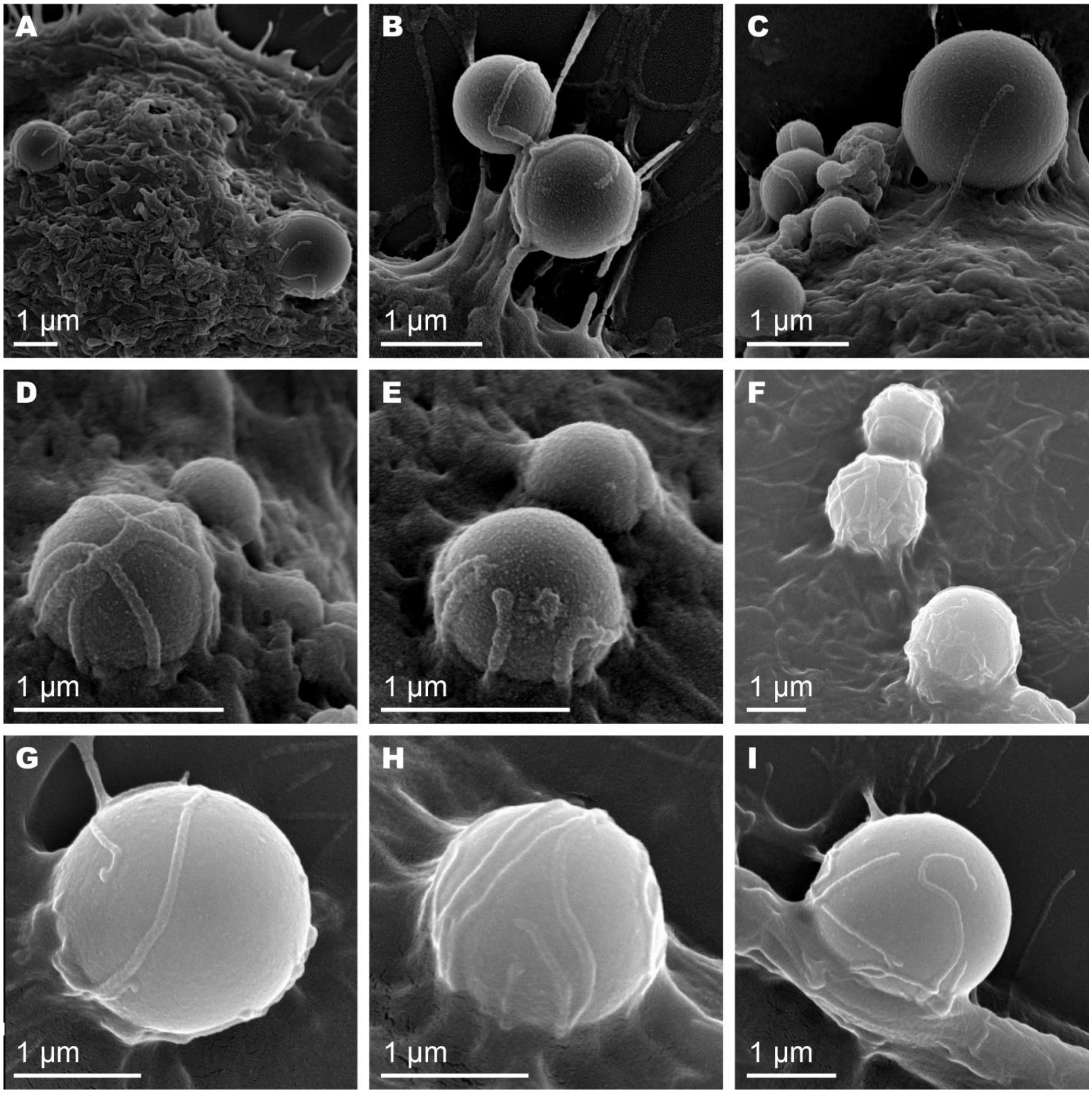

Figure S12. SEM images of coacervates representative of the "attachment/filipodia capture" stage in HeLa cells. **A-E**. HB*pep* coacervates. **F-I.** HB*pep*-SP coacervates.

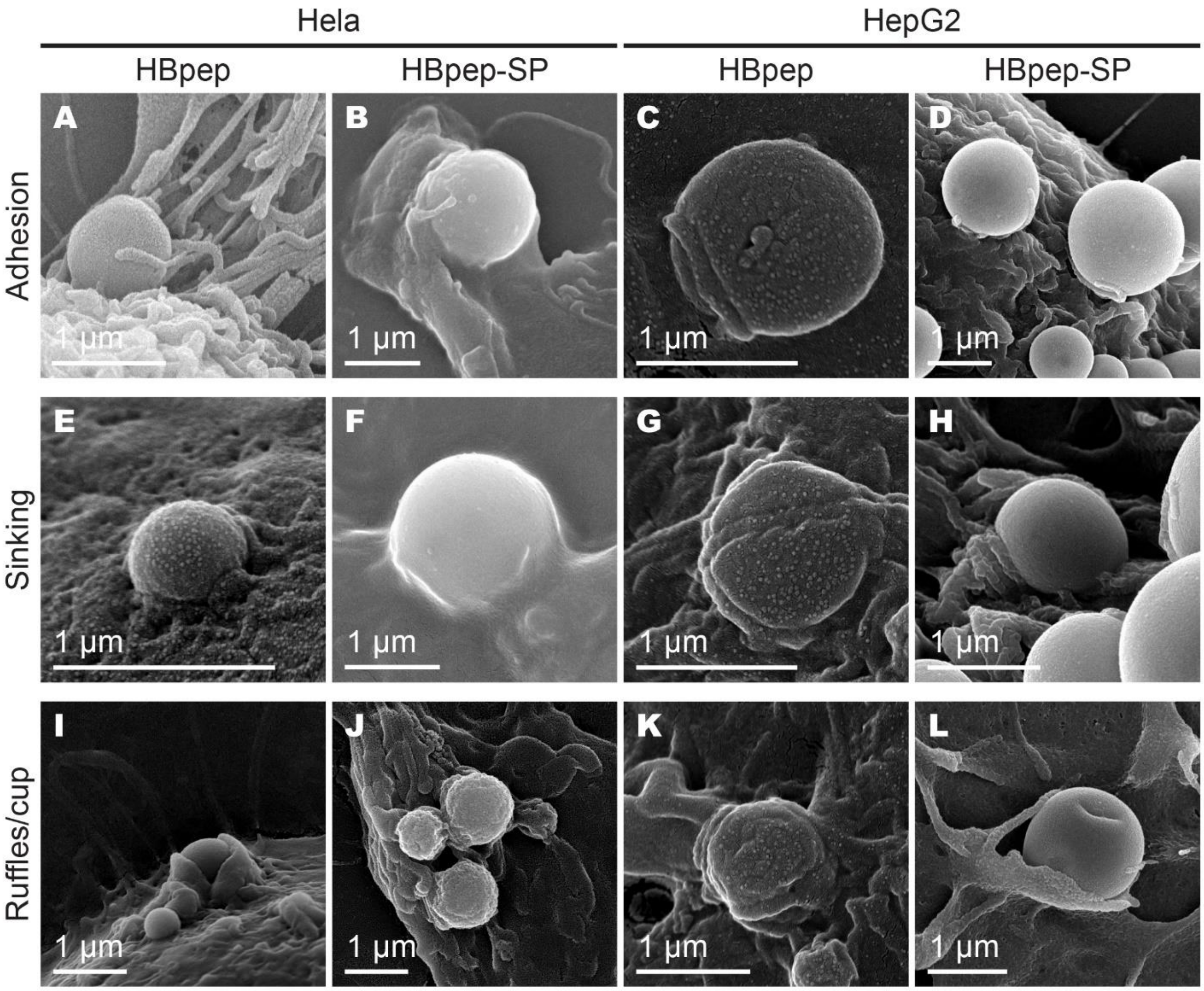

Figure S13. Comparative SEM images of Hela and HepG2 cells interacting with HBpep and HBpep-SP coacervates. **A-D**. Representative SEM micrographs of the "adhesion" stage. **E-H.** Illustrate of the "sinking" stage. **I-L**. Comparison of different ruffles/cup surrounding the coacervates across conditions.

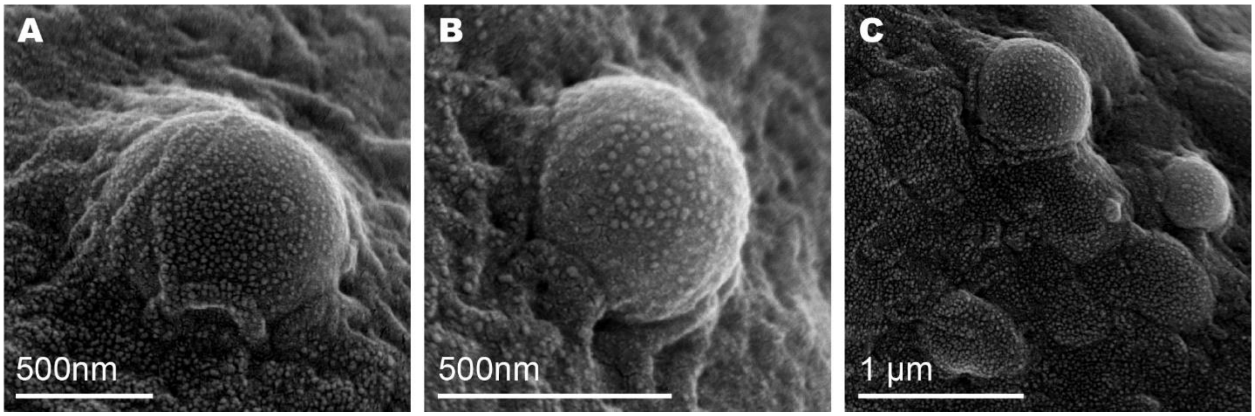

Figure S14. SEM images of coacervates representative of the “sinking” stage of HB*pep* coacervates in HeLa cell.

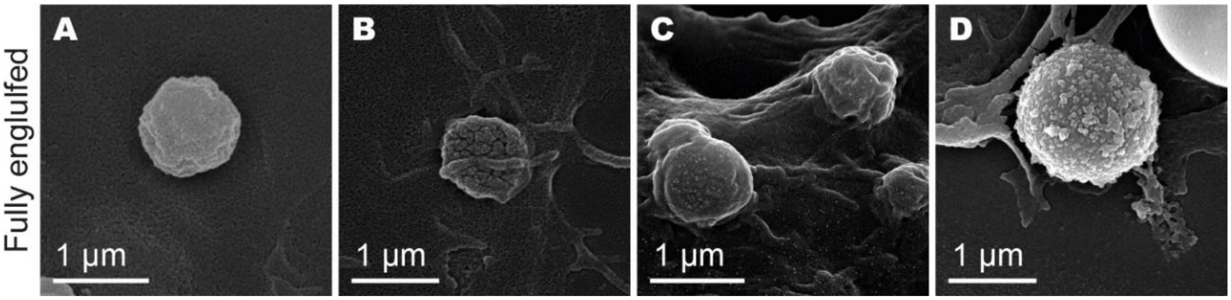

Figure S15. Representative SEM images of bumps found on the cell surface that may represent fully engulfed coacervates. **A.** HB*pep* coacervate in HeLa cell. **B**. HB*pep*-SP coacervate in HeLa cell. **C.** HB*pep* coacervate in HepG2 cell. **D**. HB*pep*-SP coacervate in HepG2 cell.

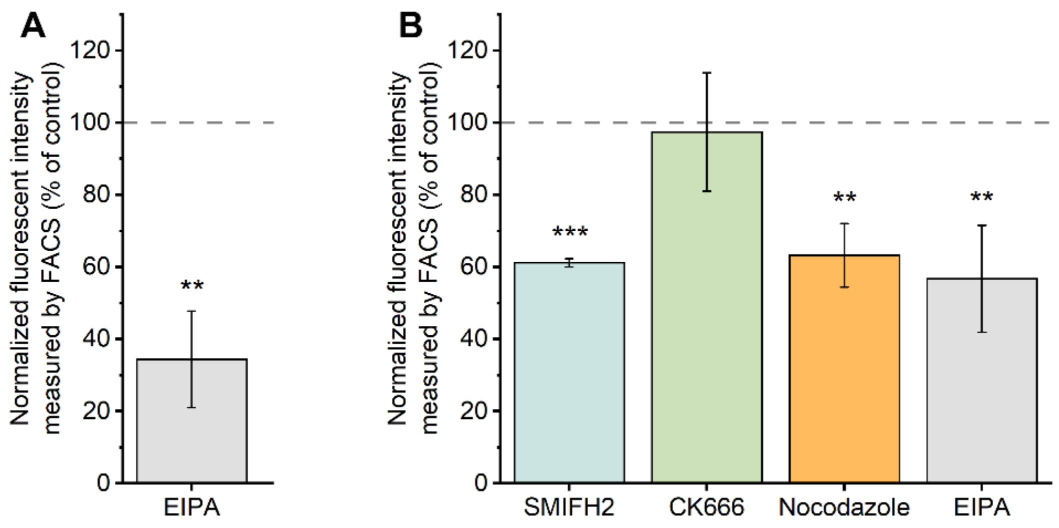

Figure S16. FACS analysis of mCherry-loaded HB*pep*-SP coacervate uptake by HeLa and EGFP-expressing HeLa- cells. **A**. EGFP-HeLa cells in the presence of EIPA (100 µM). **B**. HeLa cells in the presence of SMIFH2 (20 µM), CK666 (100 µM), nocodazole (10 µg/ml) and EIPA (100 µM). The data were normalized to control (no inhibitor added) and shown as a mean ± SD, $N = 3$. * = $P < 0.05$, ** = $P < 0.01$, ***= $P < 0.001$.

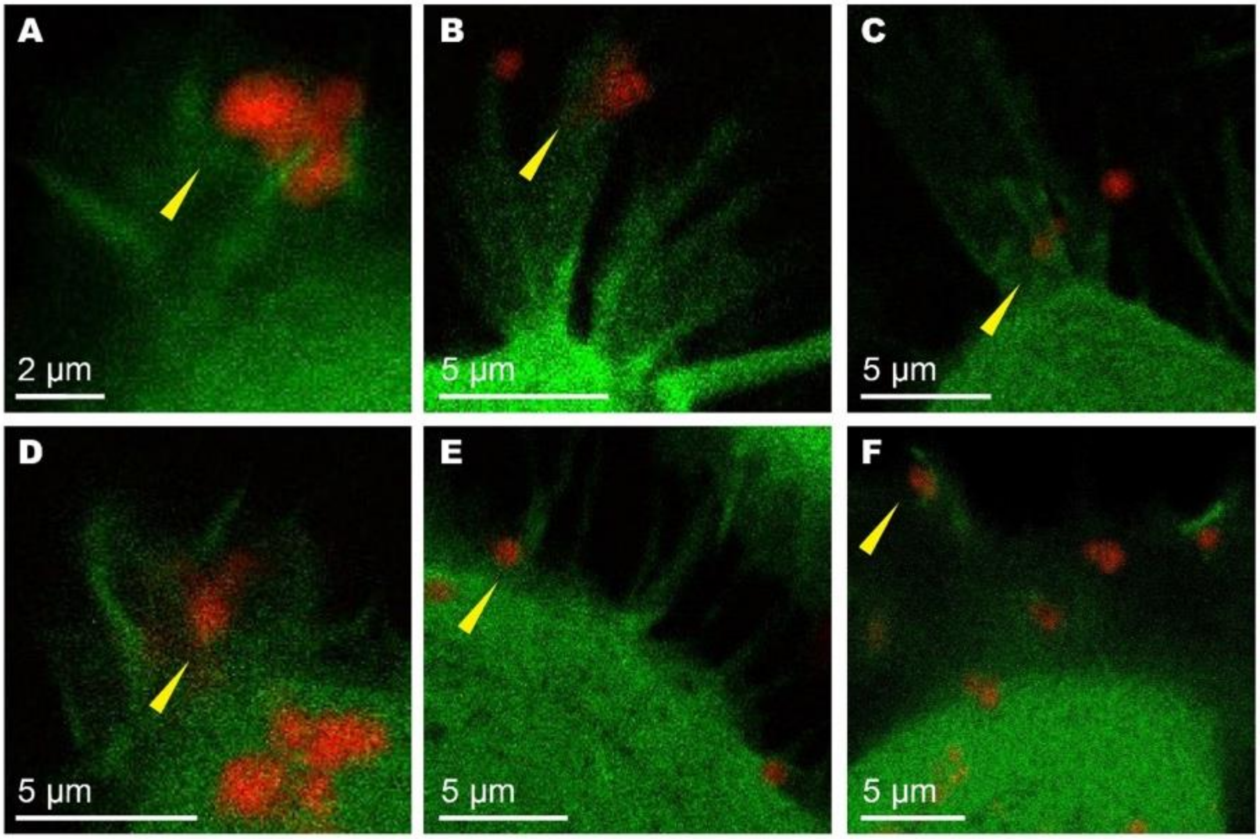

Figure S17. Fixed cell confocal imaging of HB*pep* coacervates loaded with mCherry in HeLa-GFP expressing cells. All images are representative of the "attachment/filipodia capture" stage. Capture of coacervates (red) filopodia protrusions is indicated with yellow arrows.

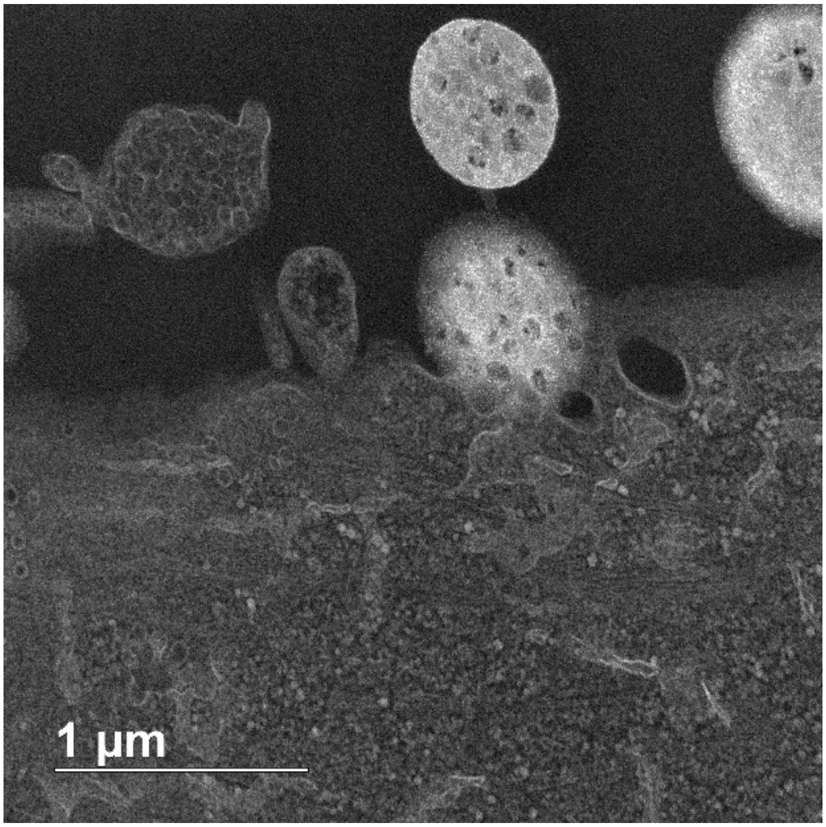

Figure S18. TEM image of HB*pep* coacervate at the HeLa cell membrane showing vesicles fusing at the uptake site.

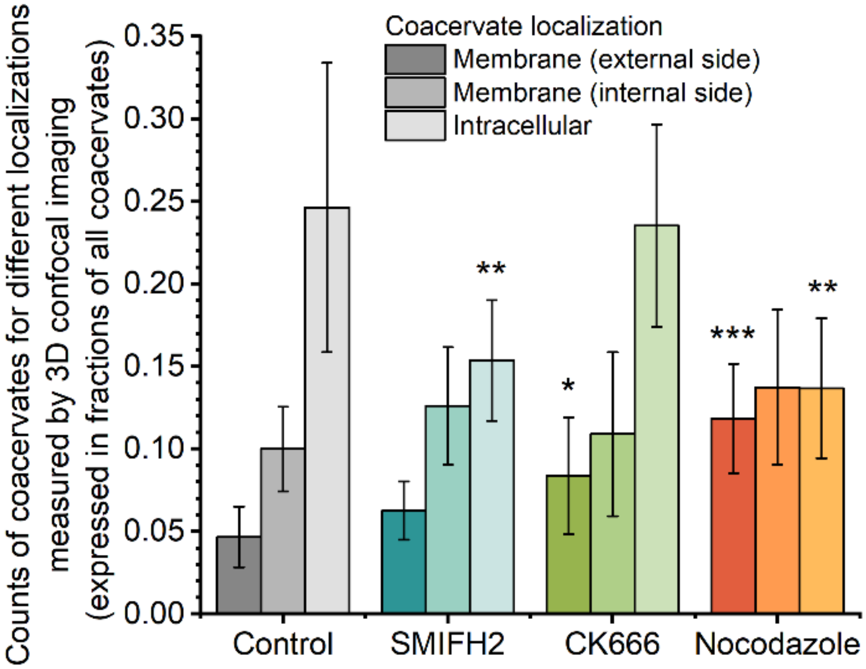

Figure S19. Coacervate counts obtained from 3D confocal imaging processed with CellProfiler to segment cell volume (using GFP channel) and coacervates (mCherry channel). All coacervates were counted in different locations: extracellular, at the membrane (external or internal side) and in the intracellular. space Counts were normalized to each sample as fractions and shown as a mean ± SD, N = 8. Counts fractions for coacervates in the extracellular space (~60%) are not shown and were 60.7% for the control, 65.8% for SMIFH2, 57.2% for CK666 and 60.7% for Nocodazole. *T*-test *p*-values are compared to the control * = *P* <0.05, **= *P* < 0.01; ***= *P* < 0.001.

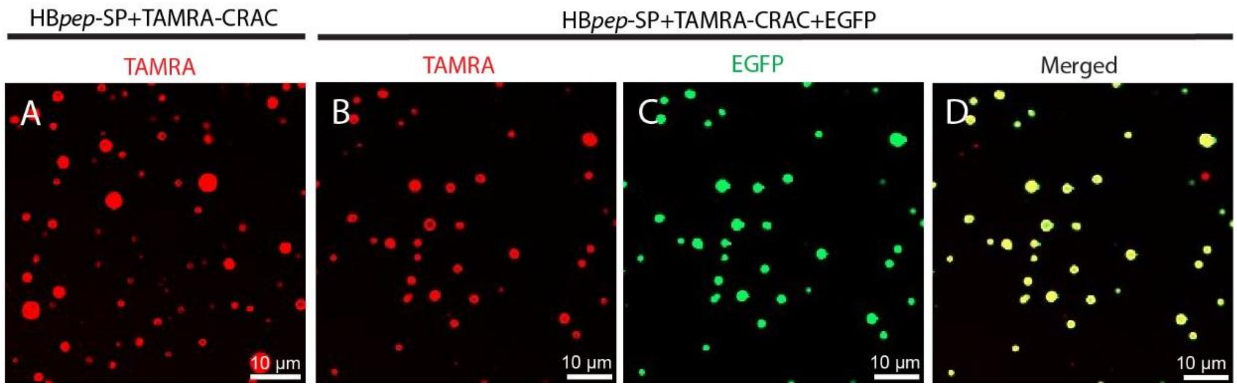

Figure S20. Confocal images of HB*pep*-SP coacervates containing **(A)** TAMRA-labelled CRAC peptide (red), or **(B-C)** TAMRA-labelled CRAC peptide and EGFP (green), demonstrating the recruitment of CRAC-peptide within the coacervates.

.

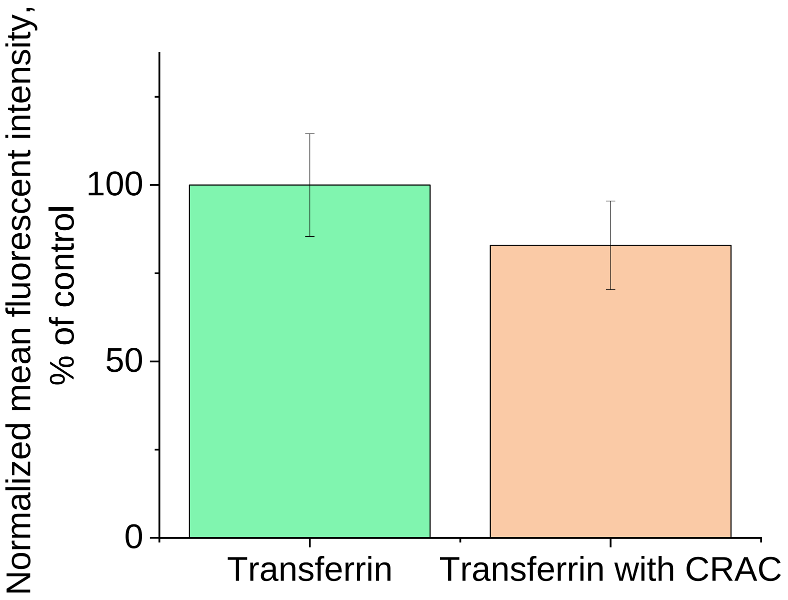

Figure S21.FACS analysis of the Alexa-488 transferrin uptake in the presence of CRAC peptide by HeLa cells demonstrated no significant changes in uptake. The data are normalized to control (no CRAC) and shown as a mean ± SD, N = 3.

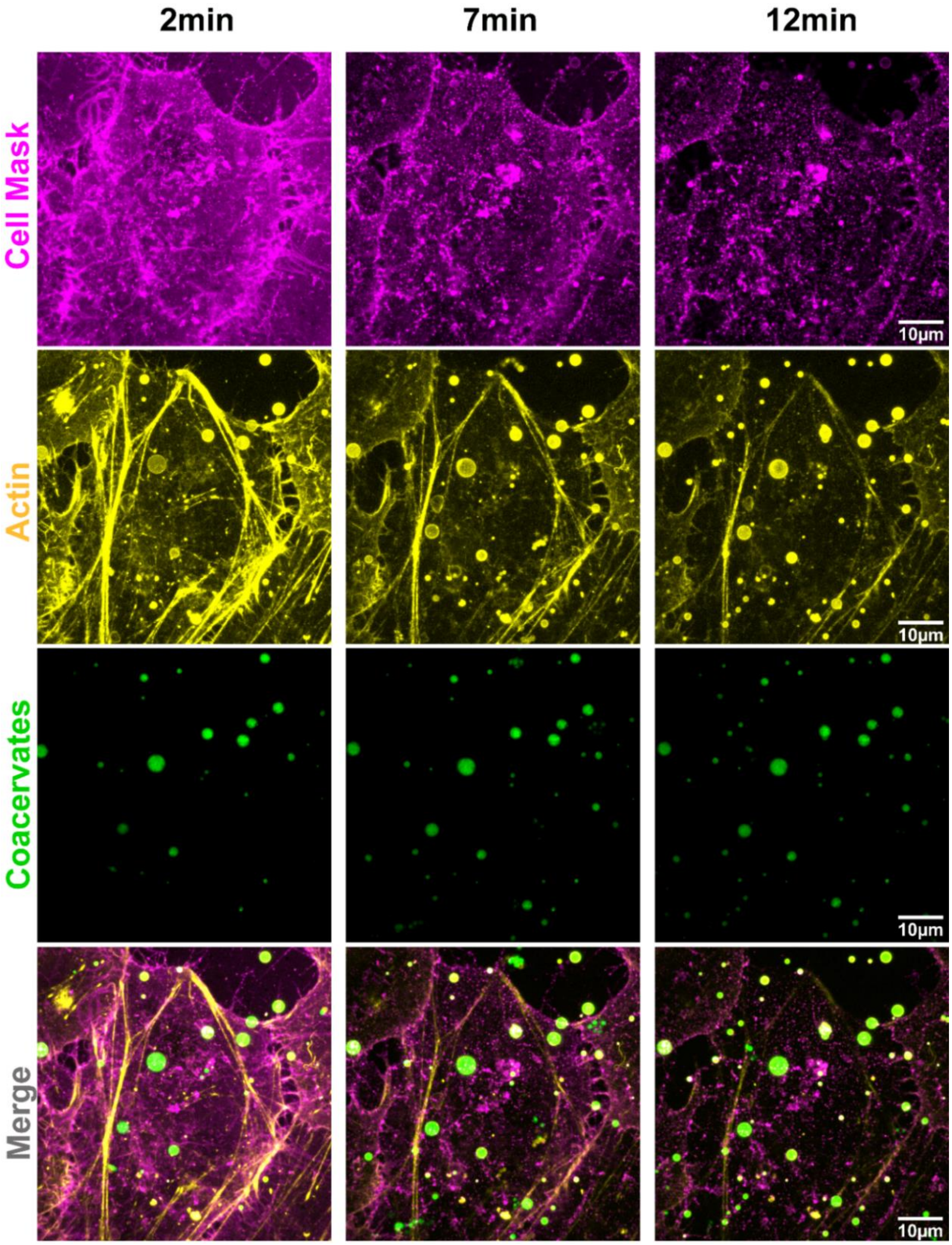

Figure S22. Live cell imaging of HB*pep*-SP coacervates uptake by HeLa cells stained with live cell actin stain. Time-lapse images of cells stained with Cell Mask Deep Red membrane dye (magenta) and ActinSpy555 (yellow) in the presence of EGFP-loaded coacervates showing that coacervate absorbed the actin dye, making colocalization experiments challenging.

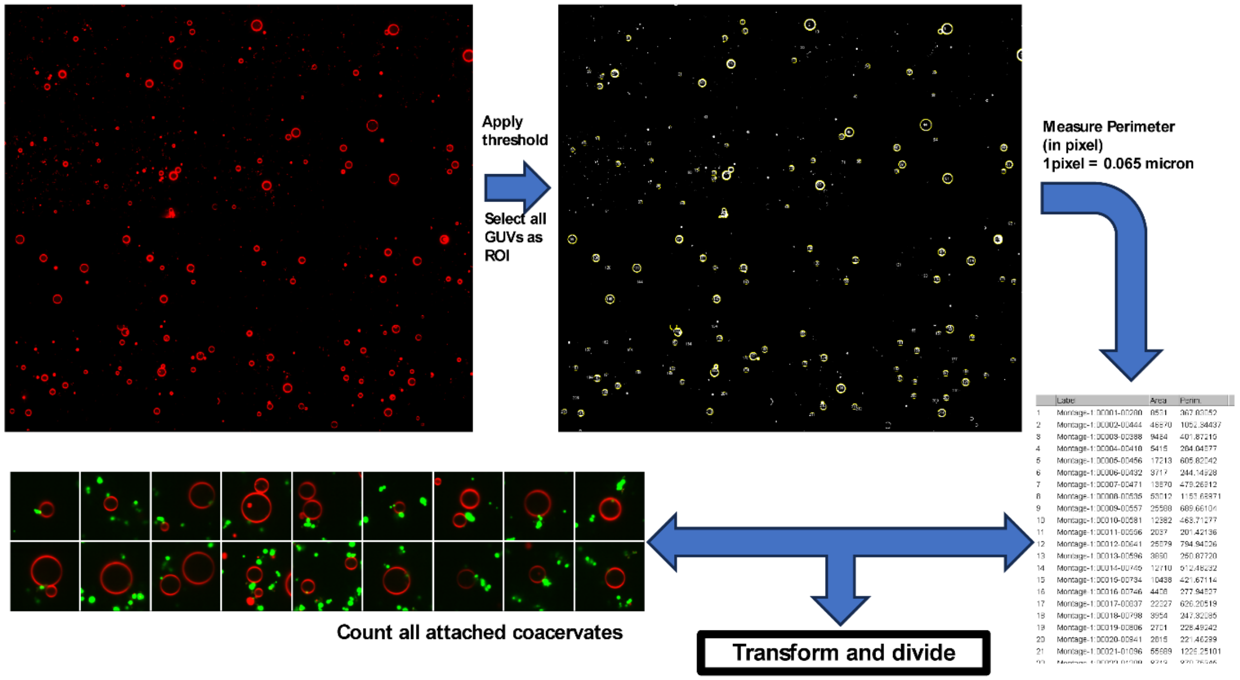

Figure S23. Schematic of the methodology used to quantify the coacervate attachment to GUVs for HB*pep*-SP coacervates using confocal microscopy. 8 fields of views were analyzed for each group. The images were subjected to processing in Image J when all GUVs were selected, and the total perimeter length was recalculated from the known pixel size. All the coacervates attachments events were carefully examined and counted, and the total number of coacervate attachments were divided by the total perimeter length of GUVs.

Table S1. Amino acid sequence of cholesterol-binding peptides (CRAC) used in the study.

I.D	Sequence	Number of Residue	Modification
CRAC	FDRARMLEEYSKRFKKFGY	19	None
CRAC scrambled	FDRARMYEKLERSFKKFGY	19	None

Table S2. Total number of GUVs analysed for the quantification of the attachment of the HB*pep*-coacervates.

GUV composition	GUV number analysed
POPC	27
PC+Cholesterol	79
POPC+POPS	68
POPC+POPS+Cholsesterol	142

Table S3. Total number of coacervates per analysed condition and average number per spot (confocal z-stack images)

Condition	Total number of coacervates analysed	Average number of coacervates per spot
Control	1785	251
SMIFH2	1838	262
CK666	4484	640
Nocodazole	3637	454

Movie S1. FIB-SEM image series of EGFP- and CRAC-peptide loaded HB*pep*-SP coacervates in HeLa cells after 3h of incubation. Disassembling coacervates are observed inside the cell.

Movie S2. Time-lapse confocal image series showing the uptake of EGFP- and CRAC peptide-loaded HB*pep*-SP coacervates in HeLa cells stained with live cell tubulin stain. The overlay of EGFP-(green) and TubulinSpy650 stain (red) image series showing the coacervates settling at the cell surface and their subsequent internalization.

Movie S3. 180-degree video of the confocal 3D reconstruction of fixed GFP-expressing HeLa cells incubated with HBpep-SP mCherry-loaded coacervates for 30 min. This shows some coacervates in the extracellular space, at the membrane and fully internalized, to be counted in CellProfiler.
